# Loss of small GTPase Rab7 activation in prion infection negatively affects a feedback loop regulating neuronal cholesterol metabolism

**DOI:** 10.1016/j.jbc.2023.102883

**Published:** 2023-01-07

**Authors:** Pearl Cherry, Li Lu, Su Yeon Shim, Vincent Ebacher, Waqas Tahir, Hermann M. Schatzl, Samia Hannaoui, Sabine Gilch

**Affiliations:** 1 Calgary Prion Research Unit, Department of Comparative Biology & Experimental Medicine, Faculty of Veterinary Medicine, University of Calgary, Calgary, Alberta, Canada; 2 Hotchkiss Brain Institute, Cumming School of Medicine, University of Calgary, Calgary, Alberta, Canada

**Keywords:** Rab7, prion, cholesterol metabolism, low-density lipoprotein (LDL), ubiquitination, BGS, bovine growth serum, BH, brain homogenate, CJD, Creutzfeldt–Jakob disease, Dil-LDL, 3,3′-dioctadecylinocarbocyanine–low-density lipoprotein, DPI, day post infection, FBS, fetal bovine serum, GEF, guanine nucleotide exchange factor, IF, immunofluorescence, LDL, low-density lipoprotein, LE, late endosome, LPDS, lipoprotein-deficient serum, PK, proteinase K, qPCR, quantitative polymerase chain reaction, RILP, Rab7 interacting lysosomal protein

## Abstract

Prion diseases are fatal and infectious neurodegenerative diseases that occur in humans and animals. They are caused by the misfolding of the cellular prion protein PrP^c^ into the infectious isoform PrP^Sc^. PrP^Sc^ accumulates mostly in endolysosomal vesicles of prion-infected cells, eventually causing neurodegeneration. In response to prion infection, elevated cholesterol levels and a reduction in membrane-attached small GTPase Rab7 have been observed in neuronal cells. Here, we investigated the molecular events causing an impaired Rab7 membrane attachment and the potential mechanistic link with elevated cholesterol levels in prion infection. We demonstrate that prion infection is associated with reduced levels of active Rab7 (Rab7.GTP) in persistently prion-infected neuronal cell lines, primary cerebellar granular neurons, and neurons in the brain of mice with terminal prion disease. In primary cerebellar granular neurons, levels of active Rab7 were increased during the very early stages of the prion infection prior to a significant decrease concomitant with PrP^Sc^ accumulation. The reduced activation of Rab7 in prion-infected neuronal cell lines is also associated with its reduced ubiquitination status, decreased interaction with its effector RILP, and altered lysosomal positioning. Consequently, the Rab7-mediated trafficking of low-density lipoprotein to lysosomes is delayed. This results in an impaired feedback regulation of cholesterol synthesis leading to an increase in cholesterol levels. Notably, transient overexpression of the constitutively active mutant of Rab7 rescues the delay in the low-density lipoprotein trafficking, hence reducing cholesterol levels and attenuating PrP^Sc^ propagation, demonstrating a mechanistic link between the loss of Rab7.GTP and elevated cholesterol levels.

Prion diseases or transmissible spongiform encephalitis are invariably fatal and infectious neurodegenerative diseases caused by the misfolding of the cellular prion protein PrP^c^ into the infectious and aggregation prone isoform called PrP^Sc^. PrP^Sc^ is the main if not the only component of prions and accumulates in the brains of infected individuals (1). This pathogenic conversion of PrP^c^ into PrP^Sc^ is triggered by a direct physical interaction between the two isoforms, inducing a conformational change in the PrP^c^ structure from a predominant alpha helical to a beta sheet–rich structure (2, 3). This results in the distinct biochemical features of PrP^Sc^ such as partial resistance to protease treatment and detergent insolubility (4, 5). Prion diseases occur in humans and other mammals and are characterized by the deposition of amyloid-like PrP^Sc^ deposits, spongiosis, astrogliosis, and progressive neuronal loss (6, 7). Human prion diseases include Creutzfeldt–Jakob disease (CJD), which is mainly sporadic in origin (8); genetic forms, *e.g.*, fatal familial insomnia and Gerstmann–Sträussler–Scheinker syndrome (9, 10); and the infectiously acquired forms of the disease such as variant CJD and Kuru (11, 12). All prion diseases are invariably fatal and have no curative treatment or prophylaxis available to date (13).

The cellular prion protein PrP^c^ is a glycosylphosphatidylinositol-anchored plasma membrane protein that localizes to lipid rafts, which are membrane microdomains rich in cholesterol (14, 15, 16, 17, 18). It has been suggested that in prion-infected cells PrP^c^ encounters PrP^Sc^ in lipid rafts, in multivesicular bodies, and in the recycling endosomes, whereby PrP^Sc^ induces the conformational change of PrP^c^ into the infectious PrP^Sc^ (19, 20, 21, 22, 23, 24). Concomitant with PrP^Sc^ accumulation, an increased expression of cholesterogenic genes has been observed in prion-infected neuronal cell lines and primary neurons, as well as in the hippocampus of preclinical mice infected with scrapie prions (25, 26). Defects in cholesterol metabolism are also reflected by an increased accumulation of cholesteryl esters in prion-infected cells and mice (27). Increased cellular cholesterol levels upon prion infection are partly attributed to a reduced cholesterol efflux from prion-infected cells, resulting from a reduced expression of cholesterol-24 hydroxylase (Cyp46A1) and an altered distribution of ATP-binding transporter cassette A1 (ABCA1) away from lipid rafts on the cell surface (28, 29). Furthermore, distinct alterations in vesicular trafficking are evident upon prion infection, including an impaired post-Golgi trafficking of proteins to the plasma membrane (30) and retrograde axonal trafficking in motor neurons of prion-infected mice (31). Enlarged early endosomes in the brains of patients with CJD (32) and the accumulation of abnormal and distorted lysosomes resulting in reduced autophagosome turnover (33) indicate that prion infection negatively affects the maturation of endosomes to lysosomes. Along the same line, previous findings from our group have highlighted that the membrane association of Rab7 is reduced in prion-infected neuronal cells, which consequently leads to a reduced lysosomal acidification and degradation (34).

Rab7 belongs to a class of small GTPases, which undergo tight regulation through an activation cycle. Rab activation is mediated by the guanine nucleotide exchange factors (GEFs), which induce a GDP to GTP exchange. Active Rab proteins exist in the GTP bound form and are tethered by prenylation to vesicle membranes, where they exert their functions by interacting with the effector proteins. Guanine activating proteins catalyze the hydrolysis of GTP to GDP, rendering Rab-GDP, which is cytosolic and inactive (35, 36).

In this study we have investigated the role of Rab7 in the upregulation of cholesterol levels because of its critical function in endocytic trafficking and cholesterol metabolism. Rab7 plays an important role in the endosome to the late endosome (LE) transition. It interacts with the Rab7 interacting lysosomal protein (RILP) and facilitates LE/autophagosome fusion with lysosomes (37) and acidification of the lysosomes (38). Rab7 also mediates the movement of lysosomes on microtubules to the positive or minus end of the cell by differentially interacting with FYVE and coiled-coil domain autophagy adaptor 1 (FYCO1) and RILP, respectively (39, 40, 41). Furthermore, it is involved in the retrograde trafficking of cargo from endosomes to the trans-Golgi network *via* its interaction with components of the retromer complex (42), as well as in the transport of cargo to the lysosomes (43, 44).

Notably, expression of a dominant-negative mutant of Rab7 inhibited the trafficking of low-density lipoprotein (LDL) to the lysosomes and its degradation (45, 46). Hence, we hypothesized that the reduced membrane association of Rab7 observed in prion infection is causally linked to the impairments in cholesterol metabolism. We found that prion infection is associated with a loss of Rab7 activation (Rab7.GTP), likely due to compromised ubiquitination, in prion-infected neuronal cell lines, primary neurons, and neurons in prion-infected mouse brains. However, the loss of Rab7.GTP is a dynamic process upon *de novo* infection of primary cerebellar granular neurons (CGNs), and cell type specific in prion-infected mouse brains. The reduction of Rab7.GTP is paralleled by an impaired lysosomal trafficking of LDL and, consequently, aberrant feedback regulation of cholesterol synthesis. Overexpression of a constitutively active mutant of Rab7 partially rescues the lysosomal transport of LDL and reduces cholesterol levels and PrP^Sc^ propagation in prion-infected neuronal cells.

Our study has portrayed the significant involvement of aberrant Rab7 function in prion infection at different stages, and the implications of its loss in function, reflected by the impairments in LDL trafficking, which culminates in an upregulated cellular cholesterol metabolism in prion-infected neuronal cells.

## Results

### Prion infection is associated with reduced Rab7 activation

**Figure 1:**
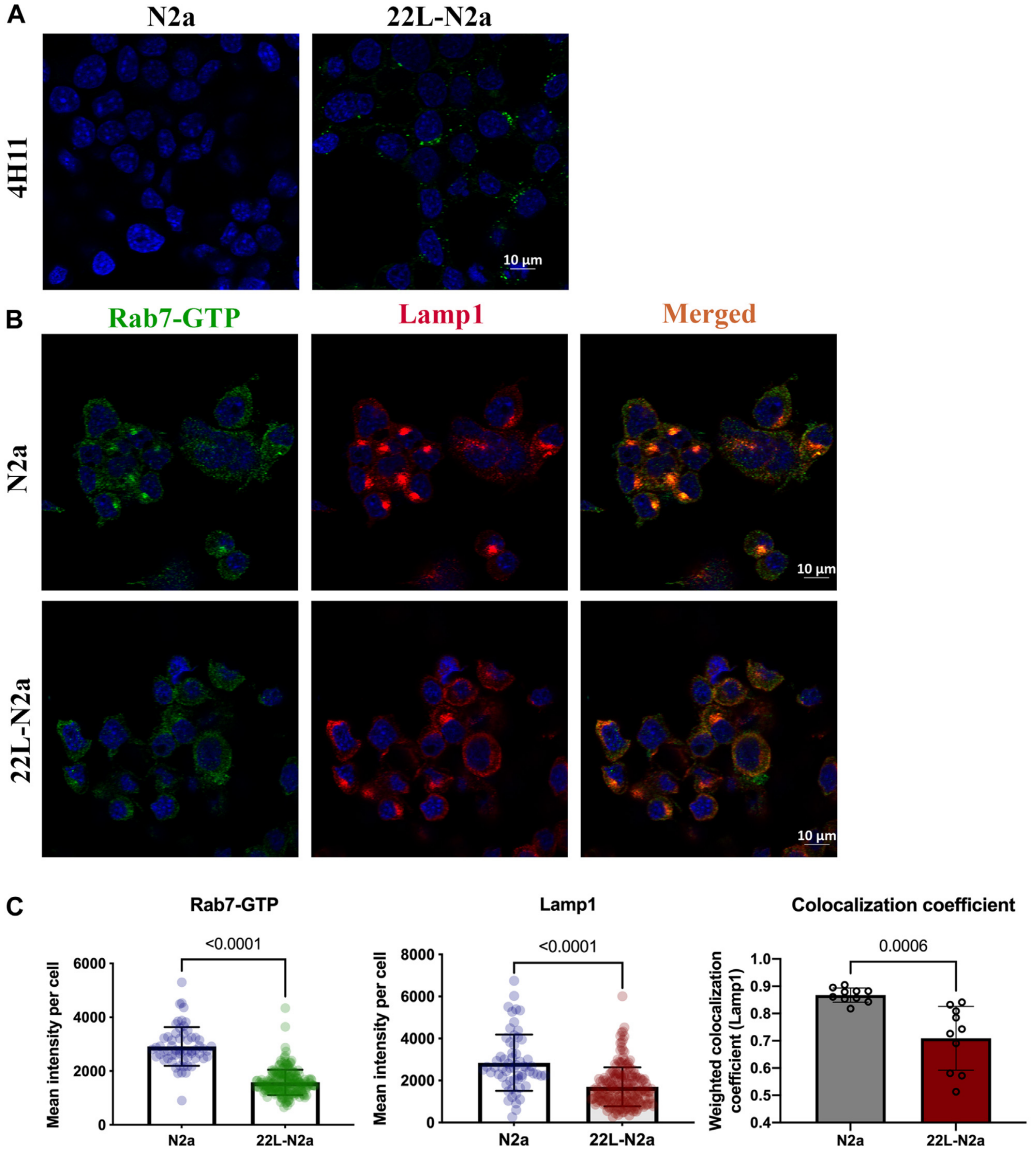
**Prion infection reduces the levels of active Rab7 in N2a cells.***A*, 22L-N2a cells were treated with 6 M GdnCl to detect PrP^Sc^ signals after probing with anti-PrP antibody (4H11). *B*, N2a and 22L-N2a cells were probed with anti-Rab7-GTP (*green*) and anti-Lamp1(*red*) antibodies and imaged using confocal microscopy. *C*, fluorescence intensity was quantified from 10 different fields of view, which approximately amounts to 300 cells per experiment. Three independent experiments have been conducted with results concurrent with the one depicted here. Unpaired *t* test was used to analyze the statistical significance between different groups, and the results are summarized in Table S1. The error bars indicate the standard deviation.

We have described previously that prion infection is associated with reduced membrane attachment of Rab7, but the total levels of Rab7 remain unaltered (34), which indicates a compromised activation status of Rab7. Hence, we quantitatively analyzed the levels of endogenous active Rab7 (Rab7.GTP) in N2a cells persistently infected with 22L prions (termed 22L-N2a) compared with uninfected N2a cells by immunofluorescence (IF) experiments. The presence of PrP^Sc^ in the 22L-N2a cells was confirmed by PrP^Sc^-specific staining after GdnCl denaturation and confocal microscopy (Fig. 1*A*). We observed a significant reduction of active Rab7 in 22L-N2a cells as compared with the uninfected cells (Fig. 1, *B* and *C*). Since Rab7 is mainly attached to late-endosomal/lysosomal membranes and is important for lysosomal maturation (43), we also analyzed the levels of lysosomal associated membrane protein (Lamp1), a common marker for lysosomes and its colocalization coefficient with active Rab7 (Fig. 1, *B* and *C*). We found that these parameters were significantly reduced upon 22L prion infection. This is in accordance with our previous findings of a defective lysosomal maturation process in prion-infected neuronal cells (34). A similar significant reduction of active Rab7 was also observed in 22L-prion-infected CAD5 cells (22L-CAD5) (Fig. S1), demonstrating that it was not a cell line–specific effect.

### Kinetics of Rab7 activation during *de novo* prion infection of cerebellar granular neurons

**Figure 2:**
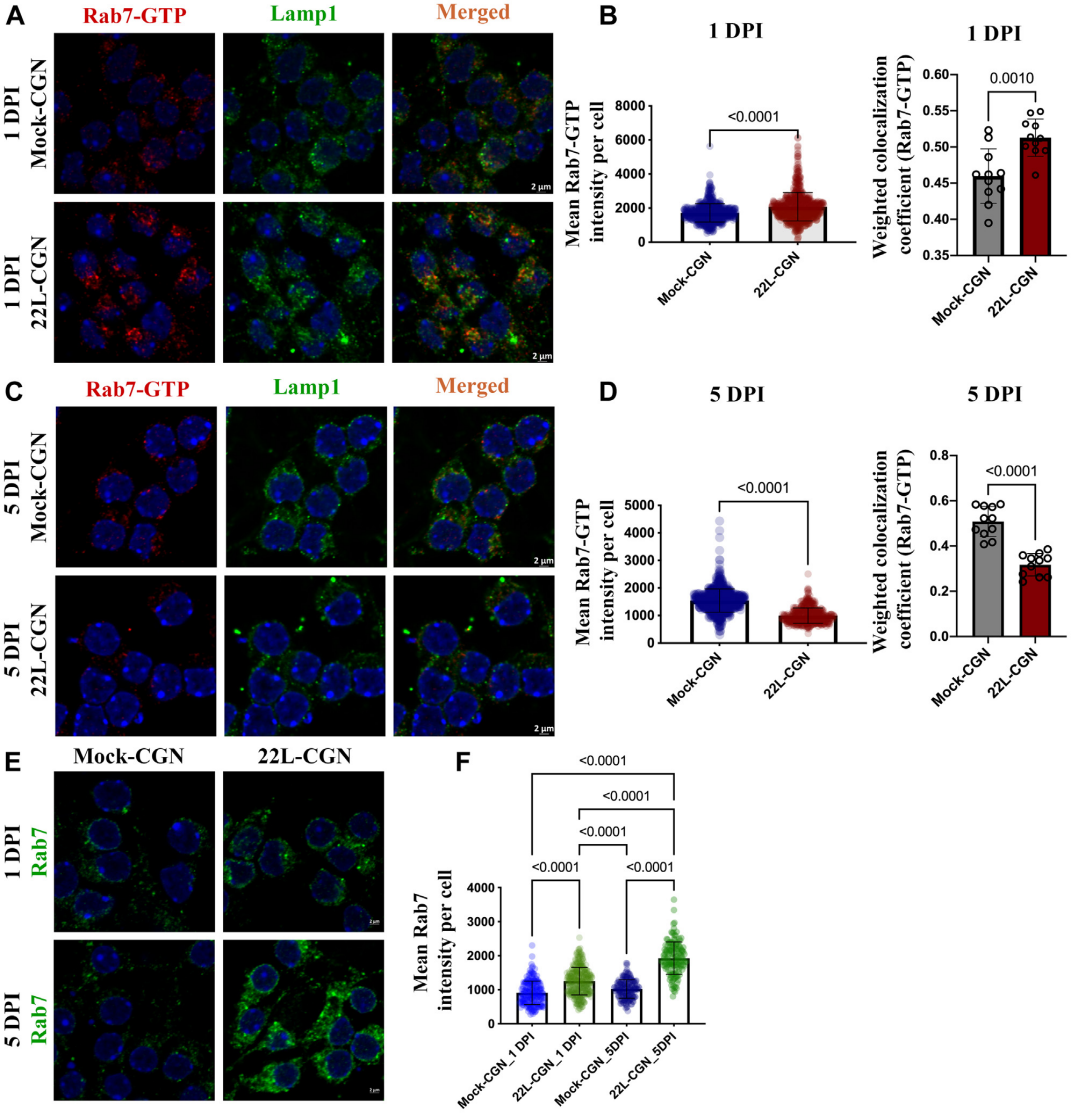
**Kinetics of Rab7 activation in *de novo* prion infection of primary cerebellar granular neurons.***A*, mock- and 22L prion-infected CGNs were probed with anti-Rab7 GTP and anti-Lamp1 antibodies and subjected to immunofluorescence experiment and confocal microscopy at 1 DPI. *B*, fluorescence intensity of Rab7-GTP and its weighted colocalization coefficient with Lamp1 at 1 DPI. *C*, mock- and 22L prion-infected CGNs were probed with anti-Rab7 GTP and anti-Lamp1 antibodies and subjected to immunofluorescence experiment and confocal microscopy at 5 DPI. *D*, fluorescence intensity of Rab7-GTP and its weighted colocalization coefficient with Lamp1 at 5 DPI. *E*, mock- and 22L prion-infected CGNs at 1 DPI and 5 DPI were probed with anti-Rab7 antibody and subjected to immunofluorescence staining and confocal microscopy. *F*, fluorescence intensity of total Rab7 levels at 1 DPI and 5 DPI. The fluorescence intensities and weighted colocalization coefficients were quantified from 10 different fields of view, which approximately amounts to 300 cells per group. Three independent experiments have been conducted with concurrent results with the one depicted here. Statistical tests used and the results are summarized in Table S1. The error bars indicate the standard deviation. To provide an overview, low-magnification images of Figure 2, *A*, *C* and *E* are shown in SF 2b, c, and e, with the boxes indicating the image area shown in here.

To study the kinetics of the loss of Rab7.GTP levels and confirm its physiological relevance, we tracked Rab7.GTP levels in response to *de novo* prion infection of CGN cultures. Successful *de novo* 22L-prion infection of CGN cultures was confirmed by the detection of PrP^Sc^ by IF assay upon GdnCl denaturation (Fig. S2*A*). A progressive increase in the intensity of PrP^Sc^ staining was observed from 1 day post infection (DPI) to 5 DPI, whereas the mock-infected CGN cultures were negative. Surprisingly, at the very early stages of infection (1 DPI), we detected a significant increase in the levels of active Rab7 and in its colocalization with Lamp1 in 22L-infected CGN cultures compared with the mock-infected control CGNs (Fig. 2, *A* and *B*). At the later stages of infection, concomitant with the increase in PrP^Sc^ levels (5 DPI), a reduction of active Rab7 and its colocalization with Lamp1 was observed (Fig. 2, *C* and *D*), consistent with our findings in the neuronal cell lines persistently infected with 22L prions. However, the overall levels of Rab7 increased from 1 DPI to 5 DPI in 22L-infected CGN, despite a loss in the levels of active Rab7 (Fig. 2, *E* and *F*). Overview images are included in Figure SF 2b, 2c, and 2d.

### Prion infection reduces Rab7 ubiquitination and effector interactions in cell culture

**Figure 3:**
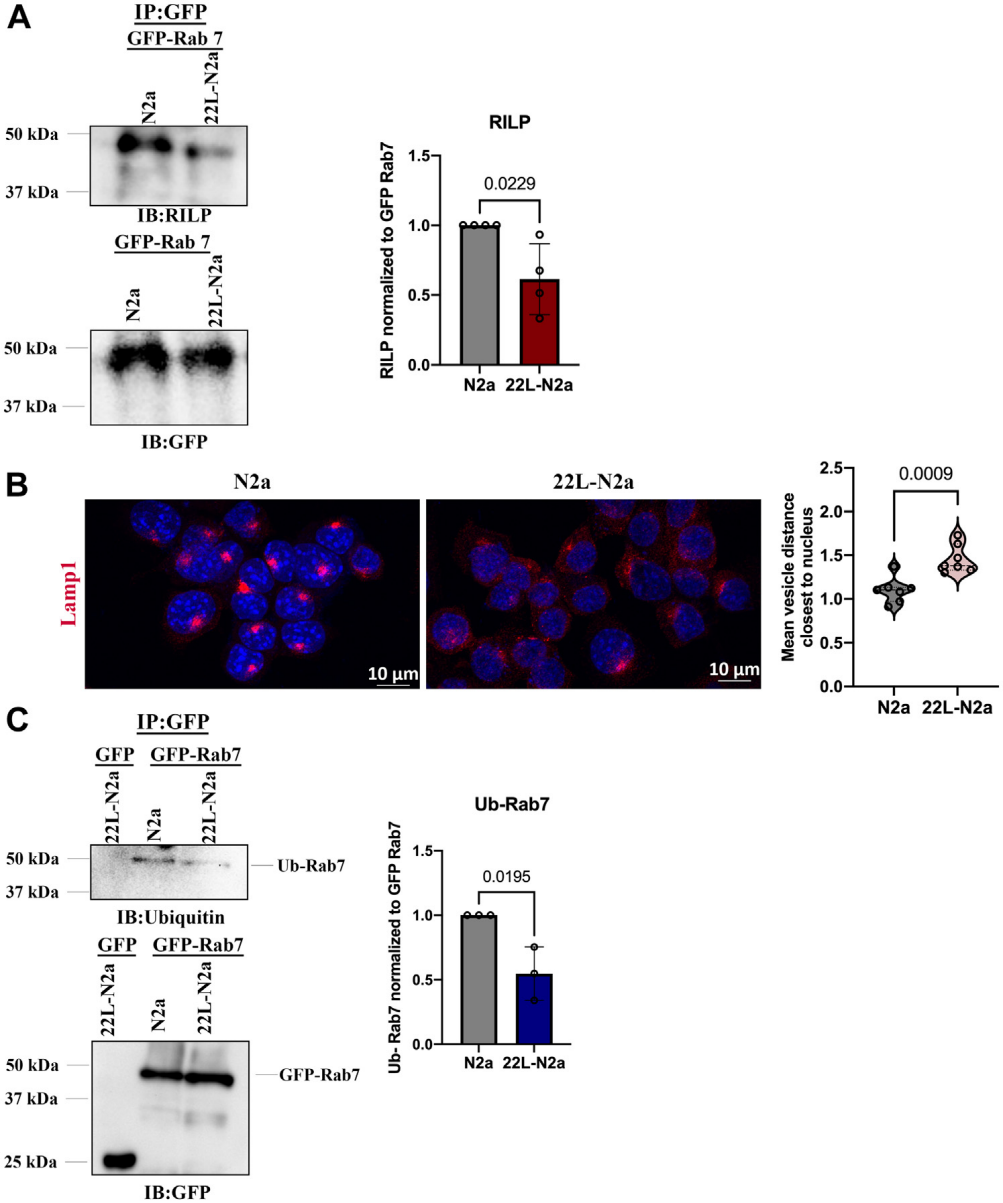
**Impaired Rab7 effector interactions in 22L-N2a cells.***A*, N2a and 22L-N2a cells were transfected with GFP-tagged WT-Rab7 and subjected to immune pull-down with anti-GFP nanobody-coupled agarose beads and subjected to immunoblotting with anti-RILP antibody. The quantitative analysis of the RILP signal normalized to the amount of GFP-tagged WT-Rab7 levels after stripping these membranes with methanol is also shown. *B*, immunofluorescence images depicting the localization of lysosomes, by probing with anti-Lamp1 antibody in N2a and 22L-N2a cells as visualized by the maximum intensity projections of the z-stack sections, obtained by confocal microscopy. All confocal images are representative of five fields of view from three independent experiments. Statistical significance was evaluated using the unpaired *t* test. The dashed and dotted lines in the violin plot depict the median and the lower and the upper quartiles of values, respectively. *C*, N2a and 22L-N2a cells were transfected with GFP-tagged WT-Rab7 and subjected to immune pull-down with anti-GFP nanobody-coupled agarose beads and subjected to immunoblotting, with anti-ubiquitin antibody. Quantitative analysis of the Ub-Rab7 signal normalized to the amount of GFP-tagged WT-Rab7 levels after stripping these membranes with methanol is also shown. Unpaired *t* test was used to analyze the statistical significance between different groups from three or more independent experiments, and the results are summarized in Table S1. The error bars indicate the standard deviation.

Next, we investigated in more detail the consequences of reduced Rab7 activation upon prion infection. We studied the interaction of Rab7 with its ubiquitous interactor RILP (39, 47) by subjecting N2a and 22L-N2a cells overexpressing EGFP-tagged wildtype Rab7 (WT-Rab7) to an immune pull-down assay. We demonstrated that the interaction of WT-Rab7 with RILP is significantly reduced in 22L-N2a cells (Fig. 3*A*). In addition, we observed an altered distribution of lysosomes in 22L-N2a cells, indicative of an impaired RILP-mediated anterograde trafficking of lysosomes to the perinuclear region (Fig. 3*B*). The levels of proteinase K (PK)-resistant PrP^Sc^ in the WT-Rab7-transfected cells were confirmed by immunoblotting (Fig. S3).

Rab7 is posttranslationally modified by ubiquitination, which is important for its membrane association and for facilitating interactions with effector proteins (48, 49). Therefore, we analyzed the Rab7 ubiquitination status upon prion infection. We observed that ubiquitination of Rab7 is significantly compromised in 22L-N2a cells (Fig. 3*C*).

### Prion infection reduces Rab7 effector interactions *in vivo*

**Figure 4:**
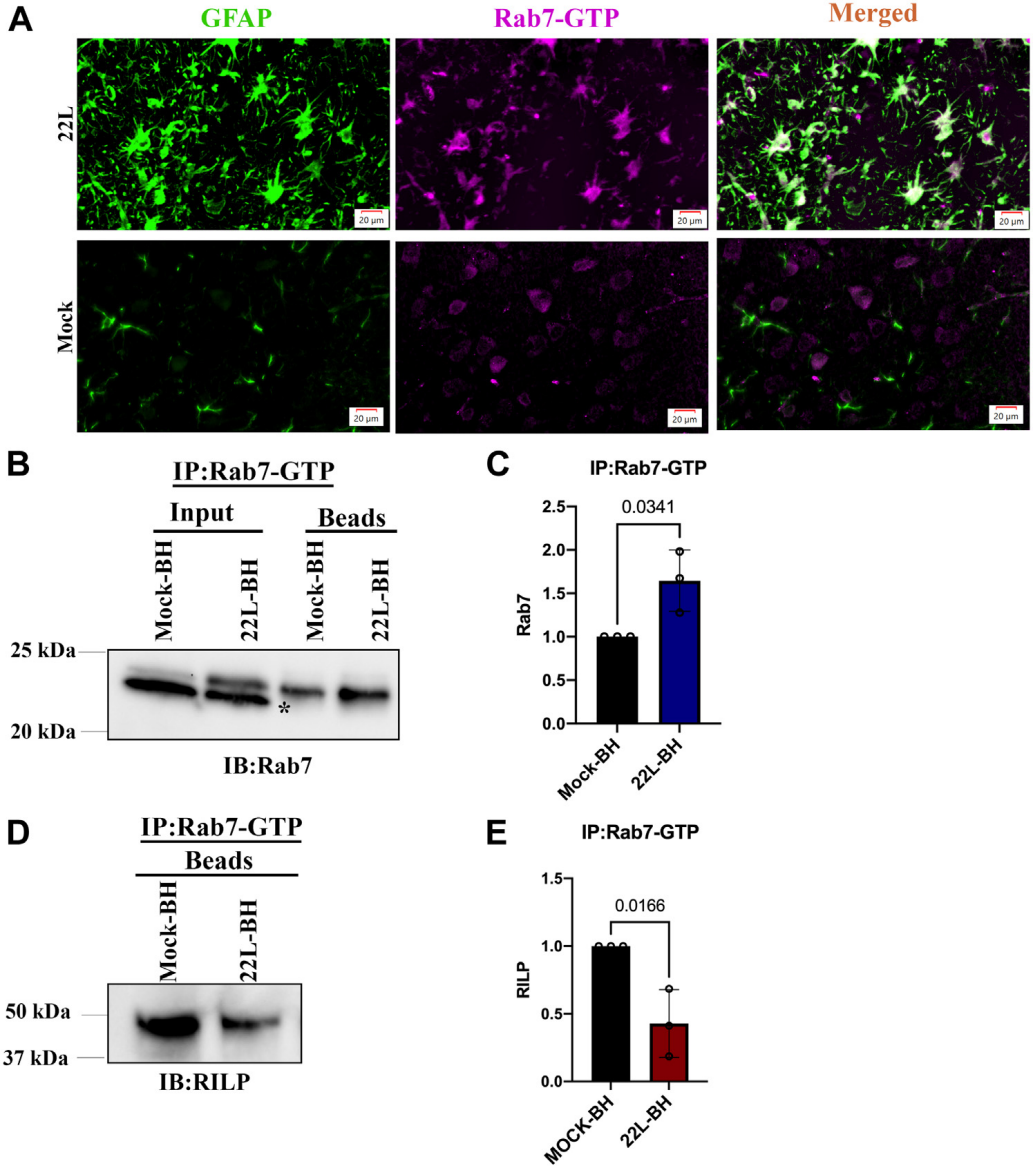
**Impaired Rab7 effector interactions in prion-infected mouse brain homogenates.***A*, brain sections from terminal mock- and 22L prion-infected mice were subjected to immunofluorescence experiments by probing with anti-active Rab7 and anti-GFAP antibodies. Mock-BH and 22L-BH (Input; 50%) were subjected to an immune pull-down assay with the anti-Rab7-GTP antibody (Beads) and probed with *B*) anti-Rab7 antibody (∗ is nonspecific band) and the signal was (*C*) quantitatively analyzed from three independent experiments. Mock-BH and 22L-BH were subjected to an immune pull-down assay with the anti-Rab7-GTP antibody and probed with (*D*) anti-RILP antibody and the signal was (*E*) quantitatively analyzed from three independent experiments. Unpaired *t* test was used to analyze the statistical significance between different groups, and the results are summarized in Table S1. The error bars indicate the standard deviation.

To verify our findings in prion-infected cell lines and primary neurons, we studied the Rab7 activation status in brains of mice infected with 22L prions harvested at the terminal stage of clinical disease and brains of age-matched mock-infected mice by IF. Surprisingly, we found an increased level of Rab7-GTP staining in GFAP-positive astrocytes in the 22L-infected mouse brains. This colocalization of Rab7-GTP with astrocytes was not observed in the mock-infected control. On the contrary, cells positive for Rab7-GTP in the control retain a neuron-like morphology, but the signal from such cells is not evident in 22L-infected brain slices (Fig. 4*A*).

Next, we performed immune pull-down assays with anti-Rab7-GTP antibody in brain homogenates of mice infected with 22L prions at the terminal stage and of age-controlled mock-infected mice. We observed an increased level of Rab7-GTP in 22L-brain homogenate (BH) with respect to the control (Fig. 4, *B* and *C*). This increased level of Rab7-GTP likely originates from astrocytes that are highly positive for Rab7-GTP (Fig. 4*A*). Also, the total level of Rab7 protein in the whole brain homogenate of the 22L-infected mice was increased, evident by the increased immune reactivity of Rab7 in the input fraction used in the immune pull-down assay, when equal amounts of mock-BH and 22L-BH were subjected to the assay (Fig. 4*B*). Despite high levels of active Rab7, its canonical interaction with RILP is significantly reduced in the 22L-BH compared with the mock-BH (Fig. 4, *D* and *E*). This suggests the existence of differential Rab7 interactors in astrocytes compared with neurons.

### The effect of prion infection in cholesterol feedback regulation

**Figure 5:**
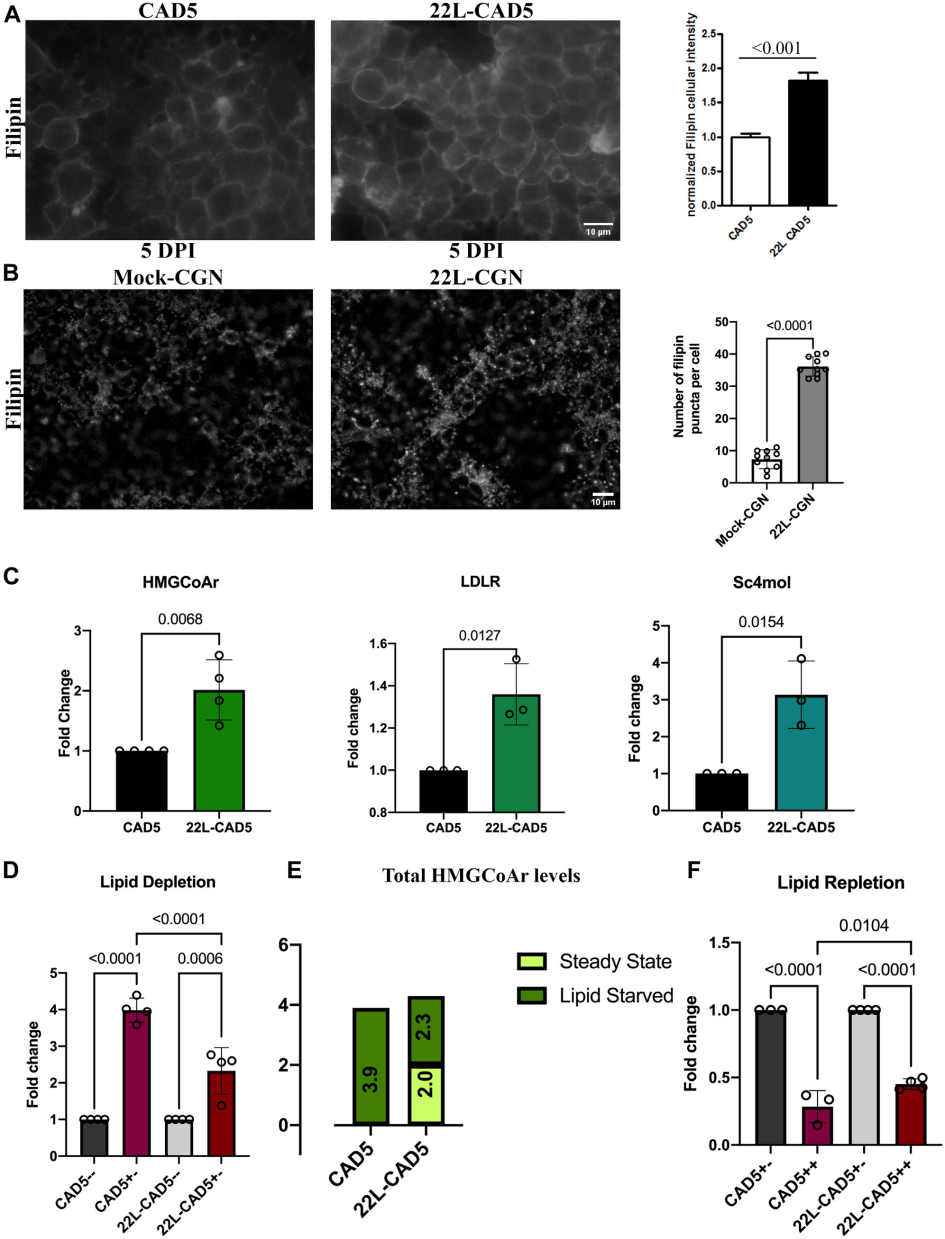
**Prion infection leads to an impaired cholesterol feedback regulation resulting in elevated cholesterol levels.***A*, CAD5 and 22L-CAD5 cells and (*B*) CGN and 22L-GGN cultures (5 DPI) were subjected to filipin staining, signals were quantified, and statistical analysis was done using an unpaired Student *t* test. *C*, gene expression analysis of the *de novo* transcription of cholesterogenic genes HMGCoAr, LDLR, and Sc4mol by RT-qPCR in 22L-CAD5 cells with respect to uninfected CAD5 cells. The gene expression levels are expressed as fold change with respect to the CAD5 cells after normalizing to the actin mRNA levels. *D*, CAD5 and 22L-CAD5 cells were grown in lipid-deficient medium for 16 h and the regulation of HMGCoAr gene expression on lipid starvation (+-), with respect to the CAD5 and 22L-CAD5 cells grown in complete medium (--), was analyzed by RT-qPCR. *E*, absolute levels of HMGCoAr gene expression on lipid-starved state in CAD5 cells and 22L-CAD5 cells. *F*, CAD5 and 22L-CAD5 cells were grown in lipid-depleted media for 16 h (+-) and replenished with 50 μg/ml of LDL for 5 h. The regulation of the HMGCoAr gene expression on lipid starvation followed by lipid repletion conditions (++), with respect to the lipid-starved CAD5 and 22L-CAD5 cells (+-), was analyzed by RT-qPCR. Statistical significance was determined from at least three or more independent experiments. Unpaired *t* test was used to analyze the statistical significance between two groups; one-way ANOVA followed by post hoc analysis using the Šídák multiple comparisons test was performed for multiple groups. The results are summarized in Table S1. The error bars reflect the standard deviation.

Prion infection is associated with elevated cholesterol levels (25, 26, 27, 28). We show that this phenotype is sustained in 22L-CAD5 cells and the *de novo* infected CGN cultures (5 DPI) at the free cholesterol levels as visualized by the filipin staining (Fig. 5, *A* and *B*) and in 22L-CAD5 cells also at the mRNA level of *de novo* cholesterogenic genes such as 3-hydroxy 3-methyl-glutaryl-coenzyme A reductase (HMGCoAr), low density lipoprotein receptor (LDLR), and sterol-C4-methyl oxidase like gene (Sc4mol) (Fig. 5*C*). In contrast to the mock-infected CGNs, the intensity of filipin staining in 22L-CGNs at 5 DPI was very distinct and more punctate at the plasma membrane enriched with free cholesterol (Fig. 5*B*). Notably, at 5 DPI we also observed a loss of Rab7 activation in 22L-CGN cultures (Fig. 2*B*). Inspired by this observation and previous studies describing the importance of Rab7 in regulating cholesterol metabolism (45, 50), we investigated the potential Rab7-mediated mechanisms leading to cholesterol accumulation upon prion infection. To this end, we analyzed the cholesterol feedback regulation in 22L-CAD5 cells upon lipid starvation and repletion conditions, compared with uninfected CAD5 cells. We observed that, upon lipid starvation (+-, Fig. 5*D*), CAD5 cells significantly upregulate HMGCoAr expression by about 4-fold compared with CAD5 cells grown in complete media, which mimics the steady-state supply of lipids (--). On the contrary, 22L-CAD5 cells upregulate the cholesterol levels only 2-fold under lipid starvation, compared with the control 22L-CAD5 cells (Fig. 5*D*). When we analyzed the absolute change in the HMGCoAr gene expression upon lipid starvation in 22L-CAD5 cells, by adding up the elevated levels of HMGCoAr at the steady state (∼2-fold; Fig. 5*C*) with that upon lipid starvation (∼2.3-fold; Fig. 5*D*), we found that it amounts to a total 4-fold increase in the HMGCoAr mRNA levels, similar to what we have observed in CAD5 cells after lipid starvation (Fig. 5*E*). This suggests that the mechanistic regulation in 22L-CAD5 cells to detect cholesterol deficits is intact. Yet, when there is a steady supply of cholesterol, 22L-CAD5 cells mimic a partially lipid-starved state, triggering *de novo* cholesterol synthesis (Fig. 5*C*). To analyze further the feedback regulation in cholesterol metabolism we supplemented the cells with 50 μg/ml LDL for 5 h after starvation. Then, the changes in HMGCoAr mRNA levels in CAD5 and 22L-CAD5 cells upon lipid repletion (++; Fig. 5*F*) were analyzed and compared with its levels at lipid starvation (+-; Fig. 5*F*). In CAD5 cells, there was an efficient feedback regulation on lipid repletion, apparent by the ∼70% reduction in HMGCoAr mRNA. On the contrary, in 22L-CAD5 cells, the feedback regulation was compromised, resulting only in an ∼55% reduction in the expression of HMGCoAr gene, significantly different from the transcriptional reduction in CAD5 cells. Hence, we conclude that the LDL-mediated feedback regulation of *de novo* cholesterogenic gene expression is impaired in 22L-CAD5 as these cells could not sense the LDL repletion as efficiently as the CAD5 cells.

### Prion infection leads to an impairment of LDL trafficking

**Figure 6:**
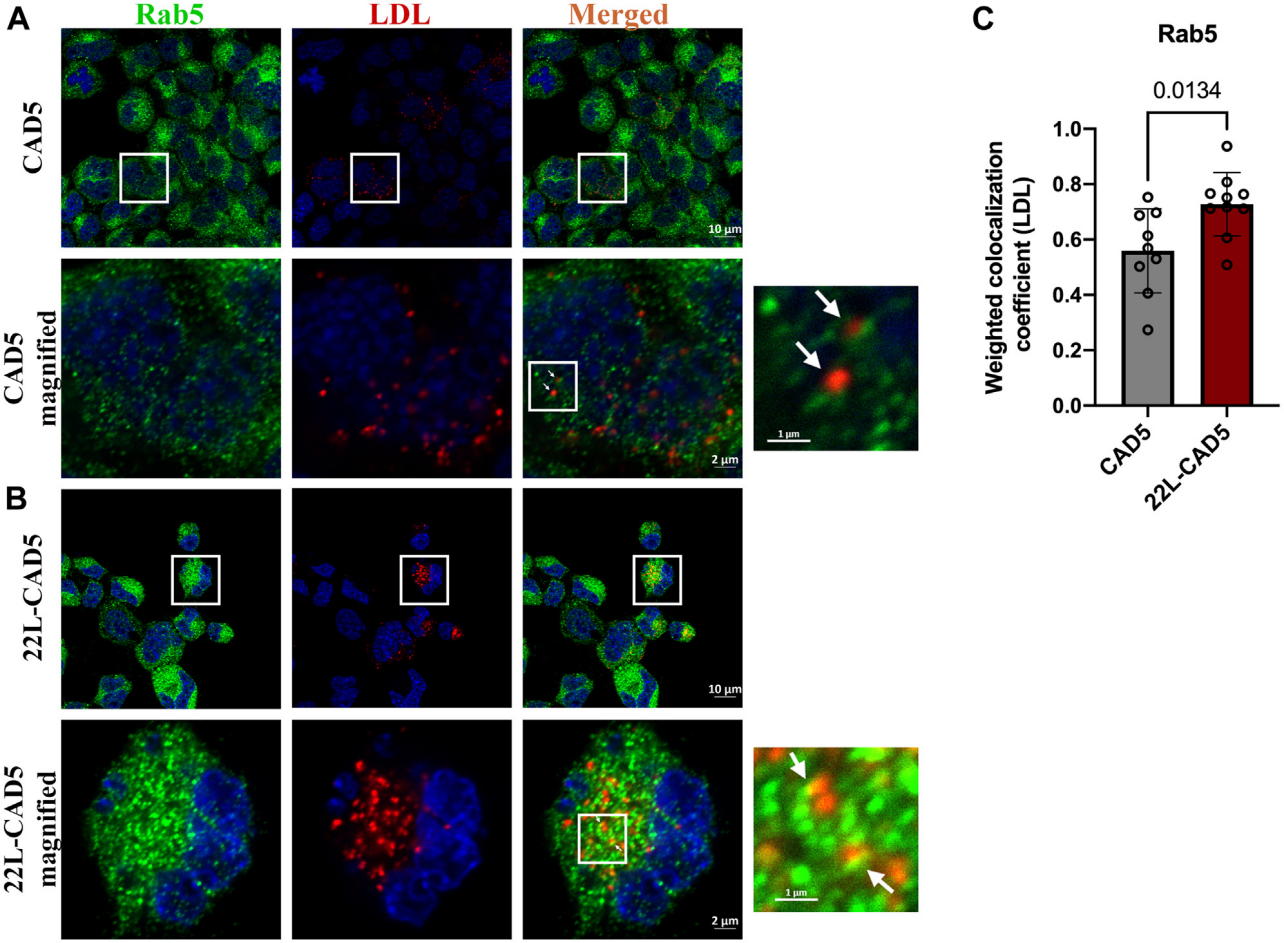
**Delayed exit of LDL from Rab5-positive early endosomes upon prion infection.** CAD5 and 22L-CAD5 cells were pulsed with 200 μg/ml Dil-LDL for 40 min at 4 °C and chased at 37 °C for 1 hour, fixed and probed with anti-Rab5 antibody, and subjected to immunofluorescence and confocal microscopy. Confocal images of LDL colocalized with Rab5 in (*A*) CAD5 cells and (*B*) 22L-CAD5 cells at the end of 1-h chase at 37 °C. *C*, quantitative analysis of the weighted colocalization coefficient of LDL with Rab5 was analyzed from 15 different fields of view, which approximately amounts to 400 cells per experiment. Three independent experiments have been conducted with concurrent results with the one depicted here. Unpaired *t* test was used to analyze the statistical significance between different groups, and the results are summarized in Table S1. The error bars indicate the standard deviation. The *arrows* indicate the localization of LDL to Rab5-positive early endosomes.

**Figure 7:**
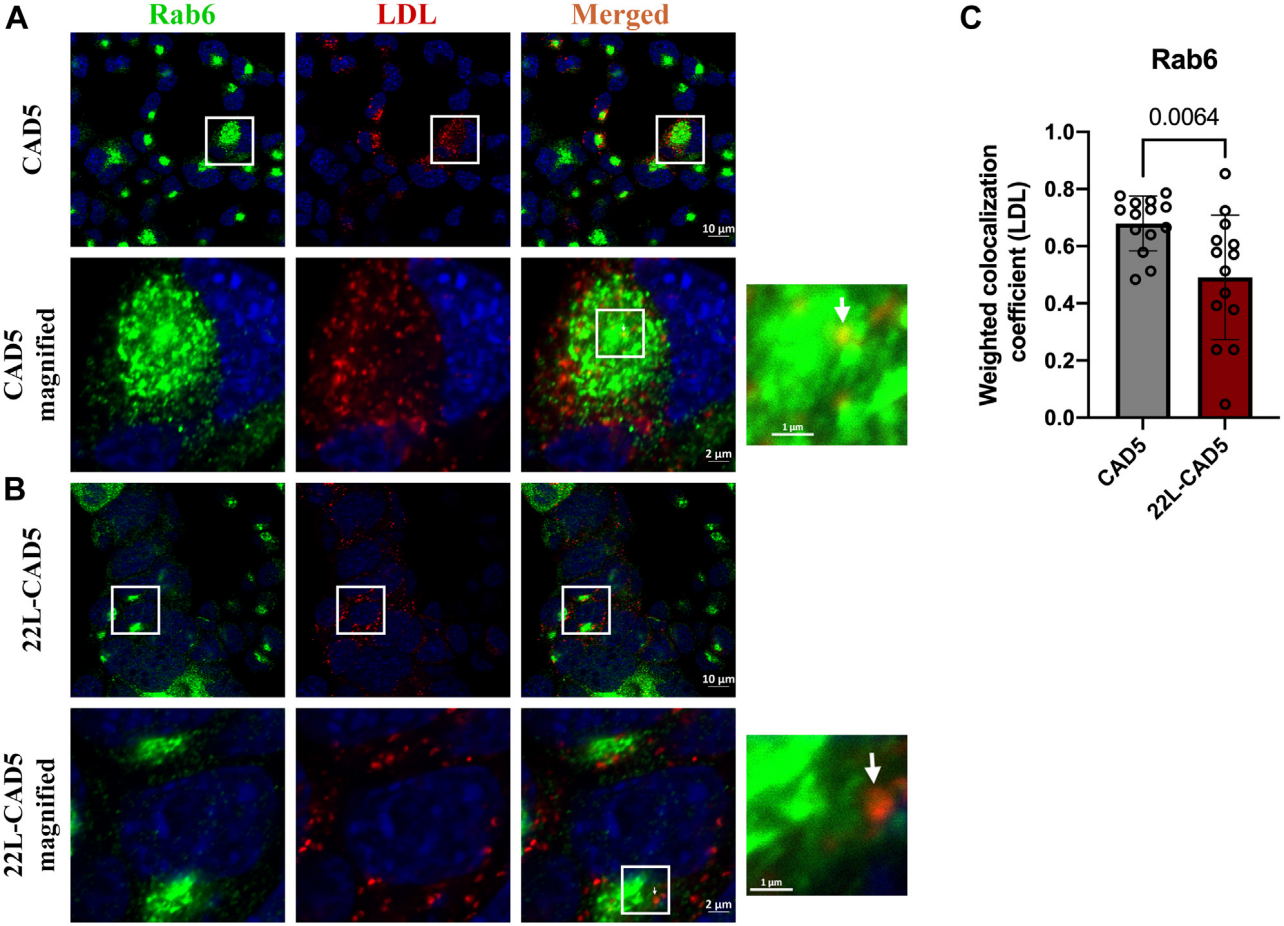
**Delayed retrograde trafficking of LDL to Rab6-positive Golgi compartment upon prion infection.** CAD5 and 22L-CAD5 cells were pulsed with 200 μg/ml Dil-LDL for 40 min at 4 °C and chased at 37 °C for 1 hour, fixed and probed with anti-Rab6 antibody, and subjected to immunofluorescence and confocal microscopy. Confocal images of LDL colocalized with Rab6 in (*A*) CAD5 cells and (*B*) 22L-CAD5 cells at the end of 1-h chase at 37 °C. *C*, quantitative analysis of the weighted colocalization coefficient of LDL with Rab6 was analyzed from 15 different fields of view, which approximately amounts to 400 cells per experiment. Three independent experiments have been conducted with concurrent results with the one depicted here. Unpaired *t* test was used to analyze the statistical significance between different groups, and the results are summarized in Table S1. The error bars indicate the standard deviation. The *arrows* indicate the localization of LDL to Rab6-positive Golgi.

**Figure 8:**
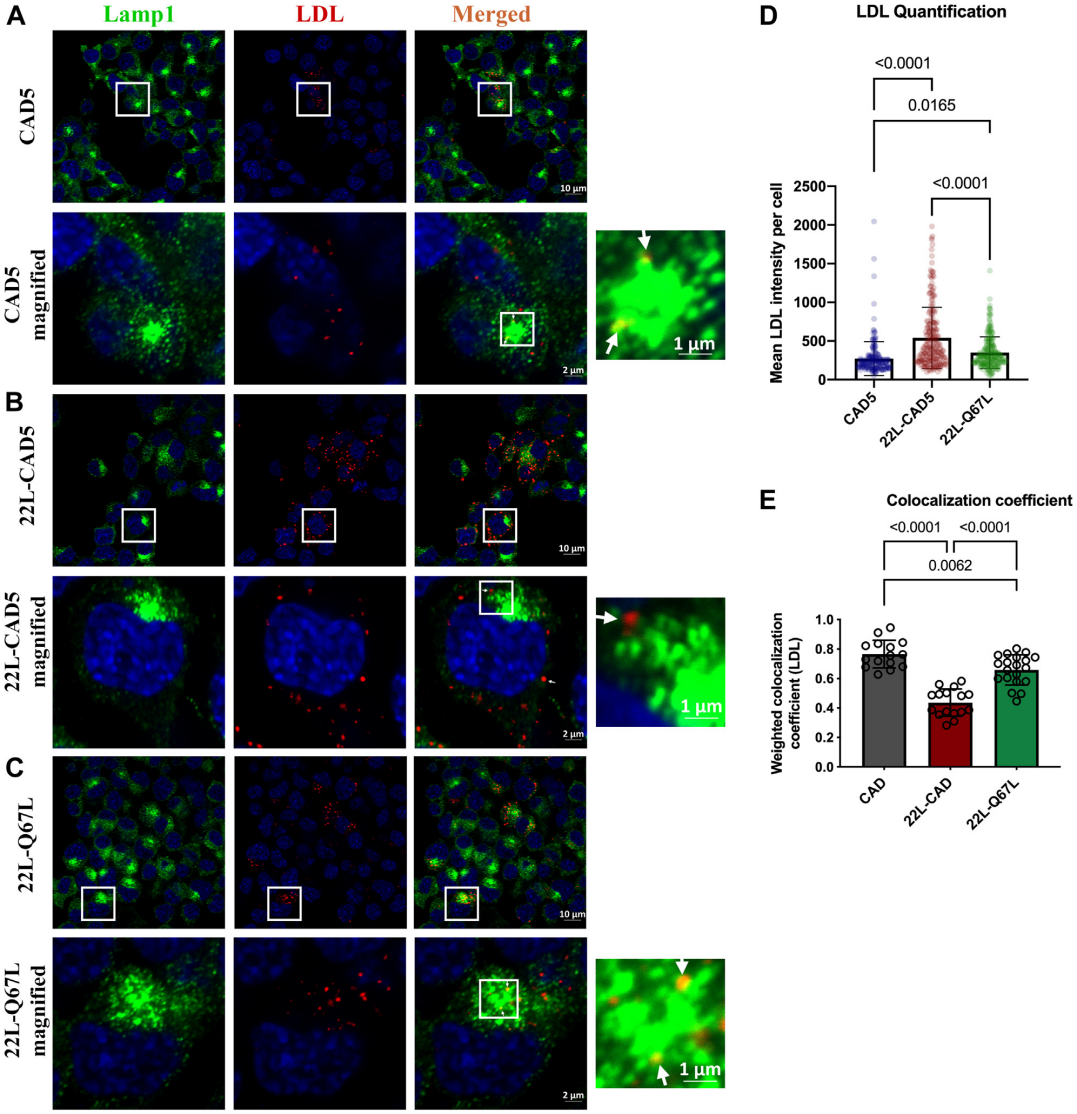
**Impaired LDL trafficking to lysosomes upon prion infection.** CAD5 and 22L-CAD5 cells were pulsed with 200 μg/ml Dil-LDL for 40 min at 4 °C, fixed, probed with anti-Lamp1 antibody, and subjected to immunofluorescence and confocal microscopy. Confocal images of LDL colocalized with Lamp1 at the end of 1-h chase at 37 °C in (*A*) CAD5 cells, (*B*) 22L-CAD5 cells, and (*C*) 22L-CAD5 cells transiently overexpressing the Rab7-Q67L mutant. Quantitative analysis of (*D*) mean LDL intensity per cell, (*E*) the weighted colocalization coefficient of LDL with Lamp1 was analyzed from 15 different fields of view, which approximately amounts to 400 cells per experiment. Three independent experiments have been conducted with concurrent results with the one depicted here. One-way ANOVA followed by post hoc analysis using Turkey multiple comparisons test was used to analyze the statistical significance, and the results are summarized in Table S1. The error bars indicate the standard deviation. The *arrows* indicate the localization of LDL to lysosomes.

To analyze the reason behind an inefficient feedback regulation of cholesterol metabolism upon repletion with LDL in prion-infected cells, we studied the trafficking of LDL to the early endosomes, the Golgi complex, and the lysosomes. We conducted pulse-chase experiments to determine the transport of Dil-LDL (3,3′-dioctadecylinocarbocyanine–low-density lipoprotein) to different compartments by analyzing its colocalization with different organelle markers such as Rab5 for the early endosomes, Rab6 for the Golgi complex, and Lamp1 for the lysosomes. The PrP^Sc^ levels in 22L-CAD5 cells were confirmed by IF assay (Fig. S4*A*). We observed an increased colocalization of Dil-LDL with Rab5 in 22L-CAD5 cells compared with the control CAD5 cells, which is indicative of a delayed exit of LDL from Rab5-positive early endosomes (Fig. 6, *A*–*C*). Since the Golgi complex (51) and the lysosomes are the two sites from where the LDL-derived cholesterol exerts its regulatory effects on cholesterol biosynthesis from the endoplasmic reticulum (ER), we analyzed the trafficking of LDL to the lysosomes and the Golgi apparatus. We found a decreased colocalization of Dil-LDL with the Rab6-positive Golgi complex (Fig. 7, *A*–C) and the Lamp1-positive late endosomal/lysosomal compartments (Fig. 8, *A* and *B*) in 22L-CAD5 cells compared with CAD5 cells, indicating an impaired trafficking of LDL to these organelles. Since Rab7 mediates the transport of LDL (45), we transiently overexpressed the constitutively active mutant of Rab7 (Rab7-Q67L) for 48 h (Fig. S4*B*) to determine whether this will rescue LDL trafficking in 22L-CAD5 cells. Rab7-Q67L-transfected 22L-CAD5 cells were subjected to pulse-chase studies with Dil-LDL, and we observed that the delay in the trafficking of LDL to the lysosomes was rescued by the overexpression of Rab7-Q67L (Fig. 8, *C* and *E*). Furthermore, we consistently observed an increased uptake of Dil-LDL in 22L-CAD5 cells compared with uninfected cells (Fig. 8, *A*, *B* and *D*). Increased LDL uptake has been shown to occur upon knockout of the Rab7-GEF C18orf8 (50). Hence, the increased uptake of LDL observed upon prion infection could be due to an impairment in Rab7 activation and is also partially rescued upon Rab7-Q67L overexpression (Fig. 8, *C* and *D*).

### Overexpression of Rab7-Q67L reduces cholesterol levels and prion propagation

**Figure 9:**
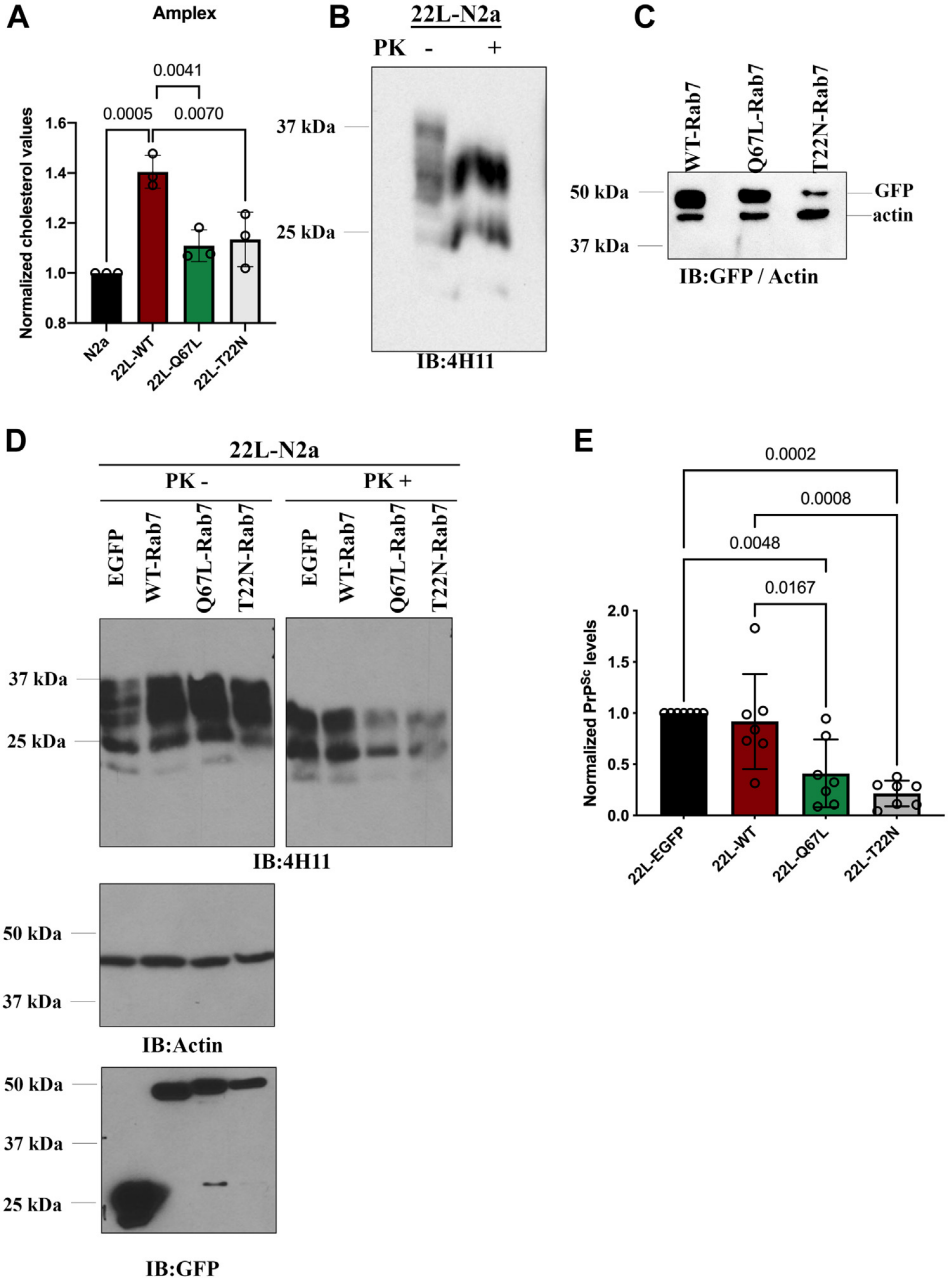
**Overexpression of active mutant of Rab7 (Q67L) reduces cholesterol levels and PrP**^**Sc**^**propagation in 22L-N2a cells.***A*, 22L-N2a cells were transiently transfected with GFP-tagged WT-Rab7 and its mutants (Q67L-Rab7, T22N-Rab7) for 48 h; cells were lysed, and the lysates were subjected to Amplex red cholesterol assay followed by the normalization of total cholesterol levels to protein concentrations. All normalized cholesterol levels are represented as a fold change with respect to the normalized cholesterol levels in N2a cells. *B*, PrP^Sc^ levels in the 22L-N2a cells were analyzed by subjecting cell lysates to PK digestion and immunoblotting with anti-PrP antibody (4H11). *C*, immunoblots depicting the transfection efficiency of different Rab7 mutants in the 22L-N2a cells. *D*, 22L-N2a cells were transfected twice in the course of 96 h with EGFP (control), GFP tagged WT-Rab7, and its mutants (Q67L-Rab7, T22N-Rab7). Cell lysates were subjected to PK digestion or not and probed using anti-PrP antibody 4H11, anti-GFP antibody, and anti-actin. *E*, quantitative analysis of the PrP^Sc^ levels in response to overexpression of WT-Rab7 and its different mutants in 22L-N2a cells. Statistical analyses were performed from three or more independent experiments using one-way ANOVA followed by post hoc analysis using the Turkey multiple comparisons test, and the results are summarized in Table S1. Error bars reflect the standard deviation.

The role of Rab7 on cholesterol levels and prion propagation was deduced upon transient overexpression of the wildtype Rab7, the constitutively active mutant of Rab7 (Rab7-Q67L), and the trans-dominant negative mutant (Rab7-T22N) in 22L-N2a cells. We conducted the Amplex red cholesterol assay to detect the total level of cholesterol in these cells (Fig. 9, *A*–*C*). We observed that overexpression of Rab7-Q67L reduced the total cholesterol levels in 22L-N2a cells, comparable with the levels of cholesterol in uninfected N2a cells. Restoring LDL trafficking (Fig. 8*C*) and hence the feedback regulation mechanism could be one way by which the active mutant exerts this effect. Overexpression of the dominant negative mutant of Rab7 also reduced the total cholesterol levels in 22L-N2a cells. However, 22L-N2a cells overexpressing WT-Rab7 retained an elevated cholesterol level, consistent with previous findings in prion-infected cells (26) and confirming a defect in Rab7 activation.

Furthermore, we showed that the effect of WT-Rab7 and its mutants on cholesterol levels correlated with the levels of PrP^Sc^ in 22L-N2a cells (Fig. 9, *D* and *E*). WT-Rab7 overexpression reduced neither the PrP^Sc^ levels nor the elevated cholesterol levels in 22L-N2a cells. Overexpression of the active mutant of Rab7 and the dominant negative mutant of Rab7 reduced the PrP^Sc^ levels in 22L-N2a cells, along with a reduction in cholesterol levels (Fig. 9*A*). Hence, we conclude that rescuing the impairment in Rab7 activation has beneficial effects in prion-infected neurons by reducing both cholesterol levels and PrP^Sc^ propagation.

## Discussion

Previous research has highlighted the importance of cholesterol, lipid rafts (52, 53, 54), and the late endosomes in the process of prion conversion (21, 22, 23, 55, 56, 57). Abrogating either the formation of lipid rafts (19, 20, 52) or late endosome maturation negatively impacts PrP^Sc^ propagation (23). In addition, it has been shown that prion infection causes an increase in cholesterogenic gene expression and free cholesterol in neurons and mouse models (25, 26) and a reduction in membrane association of Rab7 (34). Here, we demonstrate for the first time that impairments observed in cholesterol metabolism in prion-infected neurons are related to a loss in Rab7 activation.

Our study presented here on the dynamics of Rab7 activation in the context of prion infection shows that one of the initial changes that occurs in prion-infected CGN cultures is an increase in the levels of total and active Rab7 at 1 DPI. This increase in total Rab7, initially accompanied by an increase in active Rab7 is presumably a cellular response to facilitate degradation of prion protein aggregates, considering the well-documented roles of Rab7 in lysosomal maturation, cargo transport and sorting, and autophagy (37, 38, 39, 40, 41, 42, 43, 44). Results from this study (Fig. 9) and previous studies (23) have shown that expression of a dominant negative mutant of Rab7 (Rab7-T22N) interferes with prion propagation. This might indicate a role of Rab7 in facilitating prion conversion at the early stages of infection. It has also been shown that Rab7 is a direct interactor of PrP^c^ and its knockdown leads to a shift in the localization of PrP^c^ to Rab9-positive endosomes (58). Building on this information, one of the ways by which the increased levels of active Rab7 induces prion propagation might be through an increased trafficking of PrP^c^ to late endosomes, facilitating its interaction with PrP^Sc^ and hence inducing its conversion. Another possibility would be that, at early stages after *de novo* infection, an increased level of active Rab7 triggers an enhanced lysosomal activity inducing a proteolytic cleavage of the PrP^Sc^ or disaggregation of PrP^Sc^, yielding a PrP^Sc^ intermediate (seed), which is more prone to conversion. Previous studies have shown that inhibiting the proteolytic cleavage of PrP^res^, which results in an N-terminally truncated C2 fragment, by treating prion-infected cell lines with calpain inhibitors prevents PrP^Sc^ accumulation (59). However, this idea is conflicted by another study in which calpain inhibitors did not have any effect on prion propagation and hence more thorough research is required in this aspect (60). Lysomotrophic agents and cysteine protease inhibitors also reduce PrP^Sc^ propagation, again indicating the role of lysosomes/late endosomes and hence indirectly Rab7, in prion conversion (23, 61).

However, at later stages of prion infection, we observed a significant reduction in the levels of active Rab7, which negatively affects vesicular trafficking and cholesterol metabolism in prion-infected neurons. This decrease in active Rab7 is observed in prion-infected CGNs at 5 DPI, neuronal cell lines, and neurons in terminally diseased mice. In the prion-infected CGNs, it correlates with the increased accumulation of PrP^Sc^ (Fig. S2*A*) and free cholesterol levels (Fig. 5*B*), indicating a direct link with PrP^Sc^ propagation and elevated cholesterol levels. It is intriguing that the loss of active Rab7 levels occurs despite an increase in the total Rab7 pool, which sustains throughout the course of infection. This observation is in line with an earlier study demonstrating upregulation of Rab7 levels in CJD and Alzheimer disease (62). It further shows that availability of Rab7 is not a limiting factor for Rab7 activation and indicates that the activation process is impaired. PrP^Sc^ propagation occurring at 5 DPI results in the presence of PrP^Sc^ attached to host cell membranes, which might alter the membrane environment, resulting in reduced Rab7 activation. We have also shown that the ubiquitination status of Rab7 upon prion infection is reduced. Rab7 is posttranslationally modified by attachment of ubiquitin at K38 and K191. Compromised ubiquitination of K38 results in a reduced membrane association of Rab7 and decreased effector binding (48). K191 deubiquitination increases the stability of Rab7-GTP and hence its interaction with RILP (49). Since the membrane association of Rab7 (34) and its interaction with RILP are lost upon prion infection, we conclude that ubiquitination at K38 is compromised. This same phenotype is observed also in autosomal recessive early-onset Parkinson disease, indicating the existence of common mechanistic pathways in these two neurodegenerative disorders (48).

In brains of prion-infected, terminally sick mice most of the active Rab7 localizes to astrocytes. This is complemented by a study on ischemic stroke where the role of Rab7 in inducing reactive astrogliosis and glial scar is discussed. Treating the rat models of ischemic stroke with a Rab7 receptor antagonist inhibits astrogliosis and attenuates brain atrophy (63). Hence, treatment of prion-infected mice with the Rab7 receptor antagonist is a potential strategy to reduce astrogliosis and ameliorate neurological deficits associated with it. Interestingly, even though there is an overall increase in the levels of active Rab7 in the 22L-infected brain homogenate, the interaction of Rab7 with its canonical effector protein RILP is reduced.

There is an intricate association between cholesterol levels and prion propagation. First, PrP^Sc^ conversion occurs in lipid rafts, which are microdomains at the plasma membrane rich in cholesterol. Many therapeutic strategies to remediate prion propagation aimed at reducing cholesterol levels and hence reducing prion propagation (20, 29, 52, 54). Second, prion infection induces cholesterogenic gene expression and elevated cholesterol levels in neurons. Hence, another aim of this study was to elucidate whether Rab7 plays a role in the elevation of cholesterol levels observed upon prion infection (25, 26, 27, 28). In *de novo* prion-infected CGN cultures, we observed an increase in cholesterol levels at the time of the reduction of active Rab7 levels, concurrent with an increased PrP^Sc^ accumulation. We have found that 22L-CAD5 cells mimic a lipid-starved state due to a delay in the Rab7-mediated LDL trafficking from early endosomes to late endosomes/lysosomes and the Golgi complex (45), where LDL is hydrolyzed to release free cholesterol for cellular needs including feedback regulation.

The *de novo* synthesis of cholesterol is under tight regulation at the ER depending on the amount of sterols available to bind the ER resident proteins INSIG (Insulin induced genes) and Scap (SREBP [sterol regulatory element binding protein] cleavage activating protein). When oxysterols are bound to INSIGs and cholesterol is bound to Scap, SREBP cleavage in the Golgi complex and the subsequent transcription of *de novo* cholesterogenic genes are inhibited (64, 65, 66). Our results and previous studies (26) provide strong evidence for an impairment in this regulation of the cholesterol feedback in prion-infected cells. This impairment is likely a result of deficient levels of LDL hydrolyzed to free cholesterol that is made available to the ER, due to a delay in LDL trafficking and the absence of lysosomal-ER contact sites. The cholesterol generated by LDL hydrolysis is delivered to the limiting membrane of the late endosome/lysosomes by NPC2 (50) and exported across the membrane with the help of NPC1 to the ER *via* lipid-binding proteins such as oxysterol-binding protein (OSBP)-related protein 1L (ORP1L) (67), StARD3 (StAR-related lipid transfer domain-3) (68), and OSBP-related protein 5 (ORP5) (69) for feedback regulation (67). Both the export of cholesterol to the late endosome/lysosomes and cholesterol egress mediated *via* the tethering of ORP1L with the VAP (VAMP-associated proteins) at the ER membrane are Rab7-mediated processes. In the absence of active Rab7, both processes are likely compromised, and cells gain very little cholesterol from the incoming LDL. The lysosomal–ER contact sites are necessary for the transfer of cholesterol from the limiting membrane of the lysosome to the ER. Moreover, lysosome positioning we observe in prion-infected cells is similar to that reported of ORP1L knockout cells, supporting our assumption that other Rab7 effector interactions such as that with ORP1L might be reduced in prion infections (67). We also observe an increase in the uptake of LDL in 22L-CAD5 cells. This is reminiscent of the phenotype observed upon knock-down of the Rab7-GEF C18orf8 and can also be a direct consequence of the observed upregulated expression of LDLR in prion infections (26, 50). Altogether, these findings are strong evidence that the impairments in LDL metabolism observed upon prion infection are associated with the reduced levels of active Rab7. In conclusion, the upregulated synthesis of *de novo* cholesterol coupled with the increased LDL uptake due the loss of active Rab7 levels gives rise to elevated cholesterol levels upon prion infection.

We provide further evidence for a direct association between upregulation of cholesterol metabolism and reduced active Rab7 in prion infection by overexpression of Rab7-Q67L, which partially rescues the increased uptake of LDL and the trafficking of LDL to the lysosomes, thereby reducing the total cholesterol levels in prion-infected neurons. Parallel to this, we also see a reduction in cholesterol levels upon transient overexpression of the dominant negative mutant of Rab7. Even though contradictory, this observation is justified by the absence of PrP^Sc^ propagation due to the inhibition of multivesicular body maturation (23), a proposed intracellular site of prion conversion. The importance of Rab7 in reducing prion propagation has been shown by the overexpression of the active mutant of Rab7 (Rab7-Q67L). This reduction is most probably attained by enhanced lysosomal degradation and reduction of cholesterol levels; the two processes compromised upon prion infection with the loss of active Rab7. However, Yim *et al*. present conflicting results to our study in that PrP^res^ levels are not reduced upon the overexpression of active mutant of Rab7 (23). One reason for this contradiction may be based on differences in the expression levels of this Rab7 mutant, as our results have been attained at high levels of expression of this protein.

Some physiological processes mediated *via* the Rab7-RILP axis are EGFR degradation (70), multivesicular body biogenesis (70), dynein-mediated lysosomal transport and positioning (47, 71), regulation of vacuolar ATPase activity, and, hence, lysosomal acidification (38, 72). EGFR degradation and lysosomal acidification were shown to be altered upon prion infection (34). In the current study, we demonstrate that the interaction of Rab7 with RILP is reduced in 22L-N2a cells. In addition, we provide evidence that, in response to the compromised RILP interaction with Rab7, the anterograde transport of lysosomes is compromised. The lysosomal motility and distribution have critical implications in the physiology of neurons especially in the context of autophagosome clearance (73, 74, 75). Impaired autophagosome turnover has been implicated in many neurodegenerative diseases including Alzheimer disease (76, 77) and prion diseases (33, 78, 79). Lysosomal maturation is characterized by the gradual decrease in the pH of the endocytic vesicles, concomitant with the acquisition of Rab7 and Lamp1 proteins triggering anterograde movement toward the nucleus *via* association with the dynein-dynactin motor proteins (73, 80). Another factor facilitating anterograde movement of lysosomes/autophagosomes is an adequate cholesterol level through the formation of a Rab7-RILP-ORP1L tripartite complex, whereas low cholesterol conditions induce the dispersal of the lysosomes to the cell periphery (40). Hence, inefficient clearance of autophagosomes observed in prion diseases could be the result of inappropriate lysosomal positioning, maturation, and fusion with autophagosomes (75, 81, 82). It is interesting to note here that, despite the high cellular cholesterol levels in prion infection, a peripheral lysosomal distribution is observed, contradictory to previously established results (40). This is because prion infection mainly results in an increased cholesterol content in the plasma membrane, while intracellular accumulation of cholesterol in LEs as in NPC disease causes immobilization of these organelles in the perinuclear region (83).

**Figure 10:**
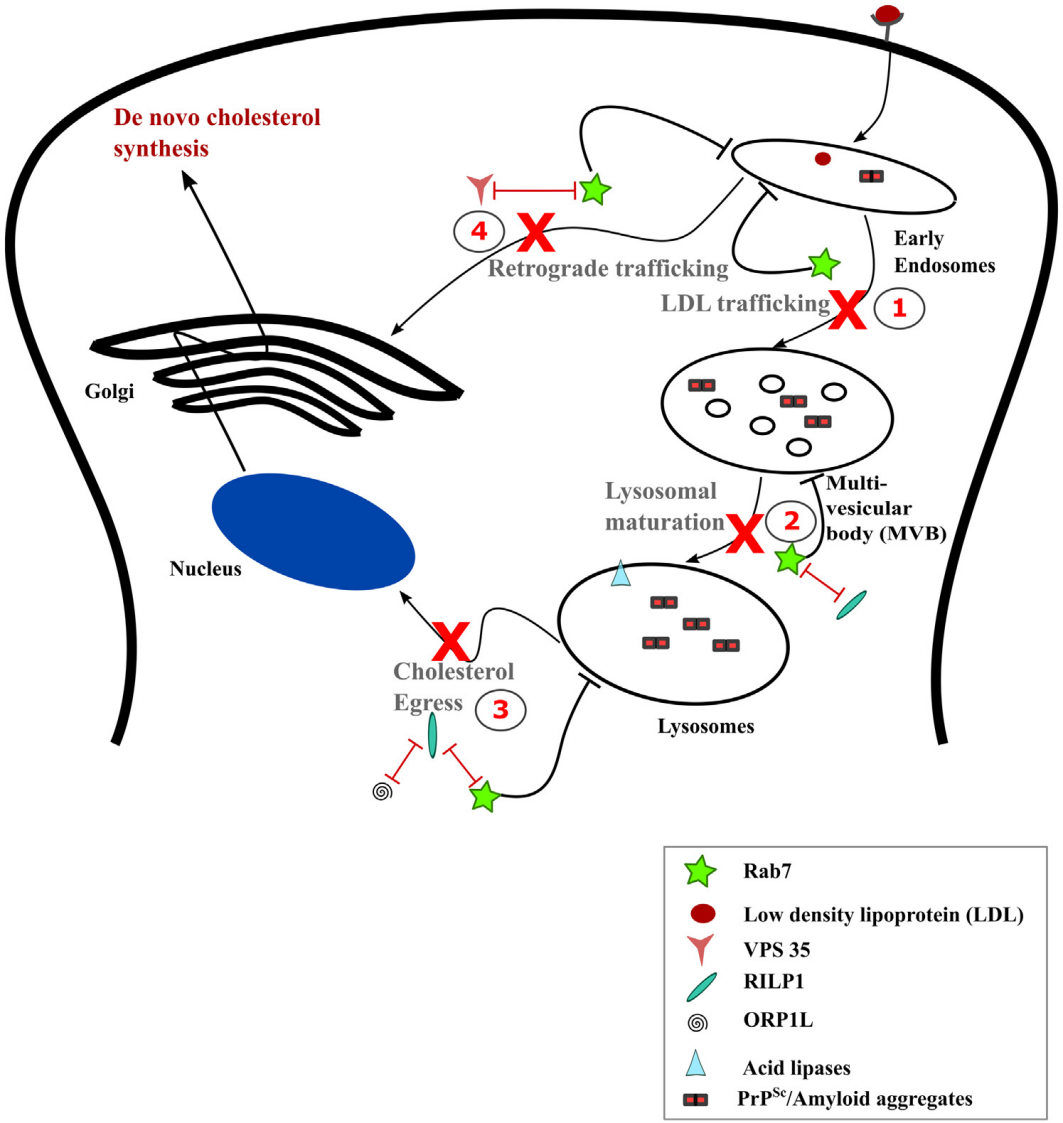
**Loss in Rab7 activation results in elevated cholesterol levels upon prion infection.** Prion infection results in a reduced activation of Rab7, which has the following consequences in an infected cell. 1. Trafficking of low-density lipoproteins (LDL) to the late endosomes or lysosomes is impaired. 2. The interaction of Rab7 with RILP is lost, which significantly impacts lysosomal transport and maturation. 3. The delay in LDL trafficking and reduced interaction of Rab7 with (Rab7 interacting lysosomal protein) RILP might consequently impact the interactions with the oxysterol-binding protein related protein 1L (ORP1L) required to facilitate cholesterol egress, which in turn mediates the feedback regulation of cholesterol to the ER resulting in *de novo* cholesterol synthesis. 4. The retrograde LDL trafficking to the Golgi complex is also affected, potentially due to a reduced interaction of Rab7 with Vps 35. Adapted from (36).

This study highlights the intricate relationship between cholesterol accumulation and prion propagation. A schematic representation of our study on how a loss in Rab7 activation can lead to elevated cholesterol levels is depicted in Figure 10. Many neurodegenerative diseases present with similar impairments associated with vesicle trafficking and lysosomal maturation defects (36). In the case of prion infections, we have demonstrated that vesicular trafficking defects due to reduced Rab7 activation can result in elevated cholesterol levels. Further studies are needed to determine whether elevated cholesterol levels are implicated in an acceleration of prion propagation and/or neurodegeneration, or whether it is a protective mechanism.

## Experimental procedures

### Ethics approval

All experiments involving animals were approved by the University of Calgary Health Sciences Animal Care Committee according to the guidelines issued by the Canadian Council for Animal Care. Mouse tissues used in the current study were derived from infection experiments published previously (84).

### Reagents and antibodies

All chemicals were purchased from Sigma unless stated otherwise. OptiMEM GlutaMAX, Dulbecco’s modified Eagle’s medium, fetal bovine serum (FBS), penicillin and streptomycin (P/S), and phosphate buffer saline (PBS) were from Gibco. Bovine growth serum (BGS) was from HyClone. Transfection reagents lipofectamine-LTX and lipofectamine 2000 were purchased from ThermoFisher Scientific. The Alexa Fluor conjugated secondary antibodies were procured from ThermoFisher Scientific and used at 1:500 concentration. Rabbit polyclonal anti-RILP (ab140188), rabbit polyclonal anti-Lamp1 (ab24170), mouse monoclonal anti-Rab7 (ab50533), and mouse monoclonal anti-EGFP (ab184601) antibodies were procured from Abcam. Rabbit monoclonal anti-Rab5 (C8B1) and anti-Rab6 (D37C7) antibodies were procured from Cell Signalling. Rabbit polyclonal anti-GFAP and mouse monoclonal anti-ubiquitin antibodies were procured from Novus Biologicals. Mouse monoclonal anti-active Rab7 (26923) antibody was procured from New East Biosciences. The PrP monoclonal antibody 4H11 used in this study has been described (85).

### Cell culture and transfections

The cell lines N2a (ATCC-CCL131; murine neuroblastoma) (26, 86) and catecholaminergic neuronal cell line CAD5 (336) were grown in Opti-MEM GlutaMAX media containing 10% FBS and 10% BGS, respectively, in the presence of 1% P/S, at 37 °C in a humidified 5% CO_2_ atmosphere. These neuronal cell lines persistently infected with the mouse adapted scrapie strain 22L (22L-N2a; 22L-CAD5) were grown under the same conditions. 22L-N2a cells were transfected using Lipofectamine-LTX reagent with pEGFP plasmids expressing EGFP-fusions with wildtype Rab7 (WT), the constitutively active mutant (Rab7-Q67L), and the dominant negative mutant (Rab7-T22 N). Briefly, 24 h and 72 h post seeding of 250,000 cells, they were subjected to transfection with 2 μg of plasmid DNA and 4 μl of LTX reagent per one 6-cm dish. After 96 h, the cells were lysed as described (26) and subjected to immunoblotting. 22L-CAD5 cells were transfected with the pCDNA3.1 myc Rab7-Q67L mutant for 48 h for the LDL pulse chase experiment described below using Lipofectamine 2000. Briefly, 100,000 cells were seeded on 18-mm coverslips in 12-well plates and were transfected with 1 μg of plasmid DNA and 2.5 μl of Lipofectamine 2000 reagent for 48 h.

### Cerebellar granular neuronal culture and *de novo* prion infection

CGN primary culture was prepared from 5- to 7-day postnatal (P5-7) newborn C57Bl/6 mice (Charles River). Cerebellum of the newborn mice were mechanically extracted and then enzymatically dissociated as described (87). CGNs were seeded at a density of 1.9 x 10^3^ cells/mm^2^ on a 12-well plastic culture plate with 18-mm sterile cover glasses coated with 10 μg/ml poly-D-lysine. Cells were then cultured in Dulbecco’s modified Eagle’s medium Glutamax I high glucose supplemented with 1% penicillin and streptomycin, 10% FBS (EuroBio), 20 mM KCl, N_2_ and B27 neuronal supplements (Gibco), and placed at 37 °C in a humidified 5% CO_2_ conditions. Prior to infection, the CGN culture is composed of 95% neurons and 5% astrocytes (87). Hence to maintain them primarily as a neuronal culture and to reduce astrocyte proliferation, 48 h post seeding, the medium was supplemented with glucose (1 mg/ml) along with the antimitotics uridine (10 μM) and fluorodeoxyuridine (10 μM).

Brains of mock- and 22L-prion-infected mice at the terminal stage of disease were homogenized in 5% (w/v) glucose. These BHs were then sonicated prior to *de novo* prion infection. The CGN cultures were infected with these BHs at a final concentration of 0.002% throughout the experiment. Samples were processed at 1 DPI and 5 DPI for IF experiments.

### Immunofluorescence and filipin staining

Cells were grown at 80% to 90% confluency on 18-mm sterile cover glasses in 12-well plates and fixed with 4% paraformaldehyde (Alfa Aesar) at room temperature (RT) for 20 min. Then they were washed thrice with PBS, permeabilized, and blocked in a solution of 10% FBS and 0.01% Triton X-100 in PBS (PBSST) for 1 hour. To detect PrP^Sc^-specific signals, cells were treated with 6 M guanidinium chloride (GdnCl) for 7 min after blocking and probed with anti-PrP (4H11) antibody as described (85). Otherwise, cells were incubated with primary antibodies (in PBSST) at their respective working concentrations for 1 hour at RT. The cells were then washed thrice in PBS and were incubated in appropriate anti-rabbit/mouse–Alexa dye coupled secondary antibodies (in PBSST) at their respective dilutions for 1 hour. Samples were washed again thrice with PBS and mounted on glass slides. Cells were imaged with a 63× oil immersion objective lens using a Zeiss LSM 700 confocal microscope. Three-dimensional z-stacks were acquired with a step size of 0.3 μm.

For filipin staining, following the fixation and the blocking steps cells were treated with 125 μg/ml filipin (Sigma) in PBSST for 2 h and imaged with a 63× oil immersion objective lens using a Leica DMI3000 B fluorescence microscope.

### Immunofluorescence staining of brain sections

Sagittal brain sections (5 μm thick) of mock- and 22L prion-inoculated FVB mice at terminal stage of disease (84) were used. Briefly, the paraffin-embedded brain sections were deparaffinized and rehydrated in sequential washes using xylene and ethanol (100%, 95%, 80%, 70%, and 0%), followed by an antigen retrieval step carried out in 10 mM critic acid buffer (pH 6.0) at high temperature (121 °C) and high pressure using an instant pot (88). The sections were cooled at RT for 30 min, washed once in TBS (10 mM Tris-HCl [pH 7.4], 150 mM NaCl) for 10 min, and blocked in a solution of 10% goat serum and 0.3% Triton X-100 in TBS for 1 h. This was followed by primary antibody incubation (rabbit polyclonal anti-GFAP (1:1500) and mouse active Rab7 antibody (1:50) in the blocking solution overnight at 4 °C in a humidifying chamber. The slides were then washed twice in TBS with 0.05% Tween 20 (TBST) and twice in TBS for 10 min each. The slides were then incubated with Alexa Fluor 488–conjugated goat anti-rabbit (1:500) and Alexa Fluor 555–conjugated goat anti-mouse secondary antibodies in the blocking solution for 1 h at room temperature followed by two washes in TBST and two washes in TBS for 10 min each. Coverslips were mounted using fluorescent mounting medium (Agilent Dako). The brain sections were imaged using an Olympus VS110-S5 virtual slide scanner at 20×.

### Lipid depletion and repletion experiments

CAD5 and 22L-CAD5 cells were grown in 6-cm dishes in complete media (OptiMEM+10% BGS+P/S) for 21 h. For lipid starvation, cells were thoroughly washed with PBS twice before seeding of 1 × 10^6^ cells in lipoprotein-deficient serum (LPDS) (Kalen Biomedicals) media (OptiMEM+10% LPDS+P/S) for 16 h. For lipid repletion, cells were replenished with LPDS media supplemented with LDL (50 μg/ml; Sigma) for 5 h. Cells grown under these conditions at 85% to 90% confluency were harvested at their respective experimental endpoints by lysing them in 1 ml trizol. Lysates were stored at −80 °C for gene expression studies using quantitative PCR (qPCR).

### RNA isolation and quantitative reverse transcriptase–PCR

RNA isolation was carried out by the organic extraction method. A volume of 200 μl of chloroform was added to the cells lysed in trizol, vortexed for about 30 s, and left at RT for 5 min for the phase separation to take place. This was followed by centrifugation at 12,000*g* for 15 min. The aqueous layer was carefully transferred to a new tube for RNA extraction. RNA was precipitated by the addition of 500 μl of isopropyl alcohol, left at RT for 10 min, and then centrifuged at 12,000*g* for 10 min. The supernatant was removed completely, and the RNA pellet was washed with 1 ml of 75% ethanol twice by vortexing, centrifugation, and removal of the supernatant. The RNA pellet was air dried and dissolved in 25 to 30 μl dH_2_O. cDNA synthesized from 1 μg of isolated RNA using Superscript II Reverse Transcriptase (Invitrogen) and random primers (Invitrogen) was subjected to quantitative PCR using Fast SYBRTM Green Master Mix (Applied Biosystems). For the qPCR cycle, samples were heated at 95 °C for 5 min and amplified for 40 cycles at 95 °C for 3 s followed by 60 °C for 30 s. The primer sequences used for the gene expression study of the cholesterogenic genes HMGCoAr, LDLR, and Sc4mol are as listed below:

5′-TTG TGA TTG GAG TTG GCA CC-3′ (HMGCoAr-F), 5′-CTC TAG GAC CAG CGA CAC AC-3′ (HMGCoAr-R), 5′-GAG TGT ATC CAT CGC AGC TG-3′ (LDLR-F), 5′-GAA TTC ATC AGG TCG GCA GG-3′ (LDLR-R), 5′-AGC CCC ACT TCC ACT GTC CA-3′ (Sc4mol-F), 5′-AAC ATG GCA GCT AAT CTT CA-3′ (Sc4mol-R). β-Actin was used as the housekeeping gene to normalize the Ct values using the following primers: 5′-CTC AGG AGC AAT GAT CTT GAT-3′ (actin-F) and 5′-TAC CAC CAT GTA CCC AGG CA-3′ (actin-R). Relative quantification of a given gene expression as a fold variation of the control was calculated using the delta–delta Ct method (89).

### Dil-LDL pulse chase experiment

CAD5, 22L-CAD5, and 22L-CAD5 cells transfected with Rab7-Q67L grown at 80% to 90% confluency were washed with PBS and then incubated with 100 μg/ml Dil-LDL (ThermoFisher) at 4 °C for 40 min. This was followed by a washing step with PBS to remove excess Dil-LDL. Then LPDS medium was added to facilitate Dil-LDL uptake. Following this pulse of Dil-LDL, cells were incubated at 37 °C for a chase period of 1 h and were washed again with PBS. Cells were then processed for IF staining and confocal microscopy as described above.

### Immune pull-down assay in cells

N2a and 22L-N2a cells grown at 60% confluency in 6-cm dishes were transfected with pEGFP plasmid (as control) and pEGFP WT-Rab7 for 48 h and lysed in 200 μl of ice-cold lysis buffer (10 mM Tris-Cl [pH 7.5], 150 mM NaCl, 0.5 mM EDTA, 0.5% NP-40, 1 mM PMSF). Lysates from four plates were pooled for one immune pull-down assay. The lysates were placed on ice for 30 min and extensively pipetted every 10 min. The lysates were then centrifuged at 17,000*g* for 10 min at 4 °C. The supernatant (∼800 μl) was transferred to a precooled tube and diluted in 300 μl of dilution buffer (10 mM Tris-Cl [pH 7.5], 150 mM NaCl, 0.5 mM EDTA) supplemented with 0.5 mM Pefabloc inhibitor. For the immune pull-down assay, agarose beads with anti-GFP nanobodies (ChromTek) were equilibrated in 500 μl ice-cold dilution buffer by centrifugation at 2500*g* for 5 min at 4 °C and the supernatant was discarded. The diluted lysate was added to the equilibrated beads and was rotated end over end for 3 h at 4 °C. The beads were then sedimented by centrifugation at 2500*g* and the flow through was discarded. The beads were washed four times in 1 ml ice-cold wash buffer (10 mM Tris-Cl [pH 7.5], 150 mM NaCl, 0.5 mM EDTA, 0.05% NP40) by centrifuging them at 2500*g* and removing the supernatant. After the last centrifugation step, the agarose beads were resuspended in 80 μl of 3× Laemmli buffer and boiled at 100 °C for 10 min. The samples were then subjected to immunoblotting. In some cases, the immunoblots were stripped with methanol for 10 min and reprobed with the required antibodies (anti-GFP) to normalize the levels of the proteins studied by immune pull-down.

### Immune pull-down assay in brain homogenate

Mock-infected (age-matched) and 22L-infected mouse brain homogenates (10% w/v) at the terminal stages of prion disease (stored at −80 °C) were prepared in lysis buffer (50 mM Tris-Cl [pH 7.5], 250 mM NaCl, 0.5% Triton X-100 and protease inhibitor cocktail [1×]) using a mechanical homogenizer (MP Biomedicals FastPrep-24 Classic Instrument [MP Biomedicals]). The lysates were centrifuged at 3000 g, and the supernatant was collected and subjected to Pierce bicinchoninic acid assay for protein quantification. Equal amounts of protein were subjected to the immune pull-down assay by overnight incubation with 2 μg of anti-active Rab7 antibody in lysis buffer in the presence of protease inhibitor cocktail. Following this, it was incubated with protein A Sepharose beads (GE Healthcare) for 3 h at 4 °C with rotation. The beads were washed with lysis buffer 5 times by centrifugation followed by the removal of supernatant prior to the final sample preparation by boiling the sample in 80 μl of 3× Laemmli buffer for 10 min. The samples were then subjected to further studies by immunoblotting.

### Cell lysis, proteinase K digestion, and immunoblotting

Cell lysis, PK digestion to detect protease-resistant PrP^Sc^, and immunoblotting were done as described (26). Cell lysis was carried out in 1 ml lysis buffer (10 mM Tris-Cl [pH 7.5], 100 mM NaCl, 10 mM EDTA, 0.5% Triton-X 100, 0.5% sodium deoxycholate), and 500 μl was subjected to PK digestion (20 μg/ml) at 37 °C for 30 min. The digestion was stopped by the addition of 0.5 mM Pefabloc inhibitor. Proteins were precipitated in methanol overnight at −20 °C, and the protein was pelleted by centrifugation at 3500 rpm for 30 min and was later dissolved in TNE buffer (Tris-Cl [pH 7.5], 150 mM NaCl, and 5 mM EDTA). The samples were then processed for immunoblotting after boiling in the appropriate amount of 3× Laemmli buffer. The remaining undigested 500 μl of cell lysate (PK-) was processed similarly and was used as a loading control by probing for actin in the immunoblotting analysis. Chemiluminescence signals (Luminata western chemiluminescent HRP substrate [Millipore Sigma]) were captured using a ChemiDoc imager (Bio-Rad) or X-Ray films (Fujifilm). Densitometric analysis was performed using Image lab software (Bio-Rad, v6.0.1) or ImageJ.

### Amplex red cholesterol assay

22L-N2a cells transfected for 48 h with pEGFP-Rab7 plasmids expressing EGFP-WT Rab7, EGFP-Rab7-Q67L, and EGFP-Rab7-T22N and untransfected control N2a cells were used. The cells were lysed as described (34) and centrifuged. The postnuclear supernatant was used for the Amplex red cholesterol assay (Invitrogen), bicinchoninic acid assay analysis, and immunoblotting to determine the transfection efficiency. Briefly, the total cholesterol levels were quantified as recommended by the manufacturer’s instructions and were normalized to the respective protein concentrations of the samples.

### Image analysis

The weighted colocalization coefficients were obtained using the Zeiss Zen image acquisition software (ZEN 2012 SP5 [64 bit]; Release Version14.0.0.0) after the intensity thresholds were set based on single probe controls. Imaris Cell 9.8 (Oxford Instruments) was used to analyze filipin staining (2D fluorescence images) and the Lamp1 3D distribution. For filipin quantification of number of puncta per cell, the nuclei and cells were defined based on the inverted filipin signal and the smoothed filipin image, respectively, and the filipin puncta segmentation was done on the original filipin image. This was necessary for accurate cell separation. For the Lamp1 3D distribution in cells, the nuclei and vesicles were segmented based on their respective images, whereas the cells were defined based on the Lamp1 images following histogram equalization. Lamp1 distribution was quantified by the mean vesicle distance from the nucleus calculated from the shortest distance of each vesicle to the cell nucleus.

### Statistical analysis

Statistical analysis was done with GraphPad Prism 9.0 software using either unpaired Student *t* test when comparing two groups or one-way ANOVA followed by post hoc analysis using the Šídák or Tukey multiple comparisons test when comparing multiple groups. Results are summarized in Table S1. Graphs were plotted using GraphPad Prism 9.0, and the bars represent mean ± SD. All experiments were conducted at least 3 times.

## Data availability

All data generated or analyzed during this study are included in this article and the supporting information.

## Supporting information

This article contains supporting information.

## Conflict of interest

The authors declare that they have no conflicts of interest with the contents of this article. The funders had no role in the design of the study; in the collection, analyses, or interpretation of data; in the writing of the manuscript; or in the decision to publish the results.
