# Supplementary material for: Loss of small GTPase Rab7 activation in prion infection negatively affects a feedback loop regulating neuronal cholesterol metabolism
Source: J Biol Chem. 2023 Jan 7;299(2):102883. doi: 10.1016/j.jbc.2023.102883 (PMC9926124; doi:10.1016/j.jbc.2023.102883)
Supplement: Supplemental data [file mmc1.pdf]

Supplementary Information

Loss of small GTPase Rab7 activation in prion infection negatively affects a feedback loop regulating neuronal cholesterol metabolism

Pearl Cherry[1,2], Li Lu[1,2], Su Yeon Shim[1,2], Vincent Ebacher[2], Waqas Tahir[1,2], Hermann M Schatzl[1,2], Samia Hannaoui[1,2], Sabine Gilch[1,2]

[1] Calgary Prion Research Unit, Department of Comparative Biology & Experimental Medicine, Faculty of Veterinary Medicine, University of Calgary, Calgary, Alberta, Canada.

[2] Hotchkiss Brain Institute, Cumming School of Medicine, University of Calgary, Calgary, Alberta, Canada.

Supplementary figures and figure legends

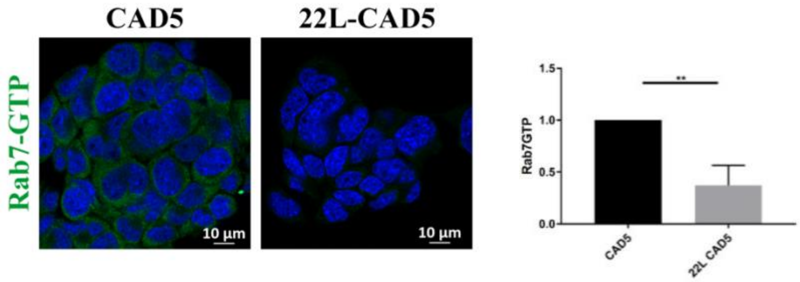

Figure SF1: 22L-prion infection reduces the levels of active Rab7 in CAD5 cells. CAD5 and 22L- CAD5 cells were probed with anti-Rab7-GTP antibody and fluorescence intensity was quantified from 10 different fields of view per group from 3 independent experiments. Unpaired t-test was used to analyze the statistical significance between different groups and the error bars indicate the standard deviation. (**, *p* values <0.01).

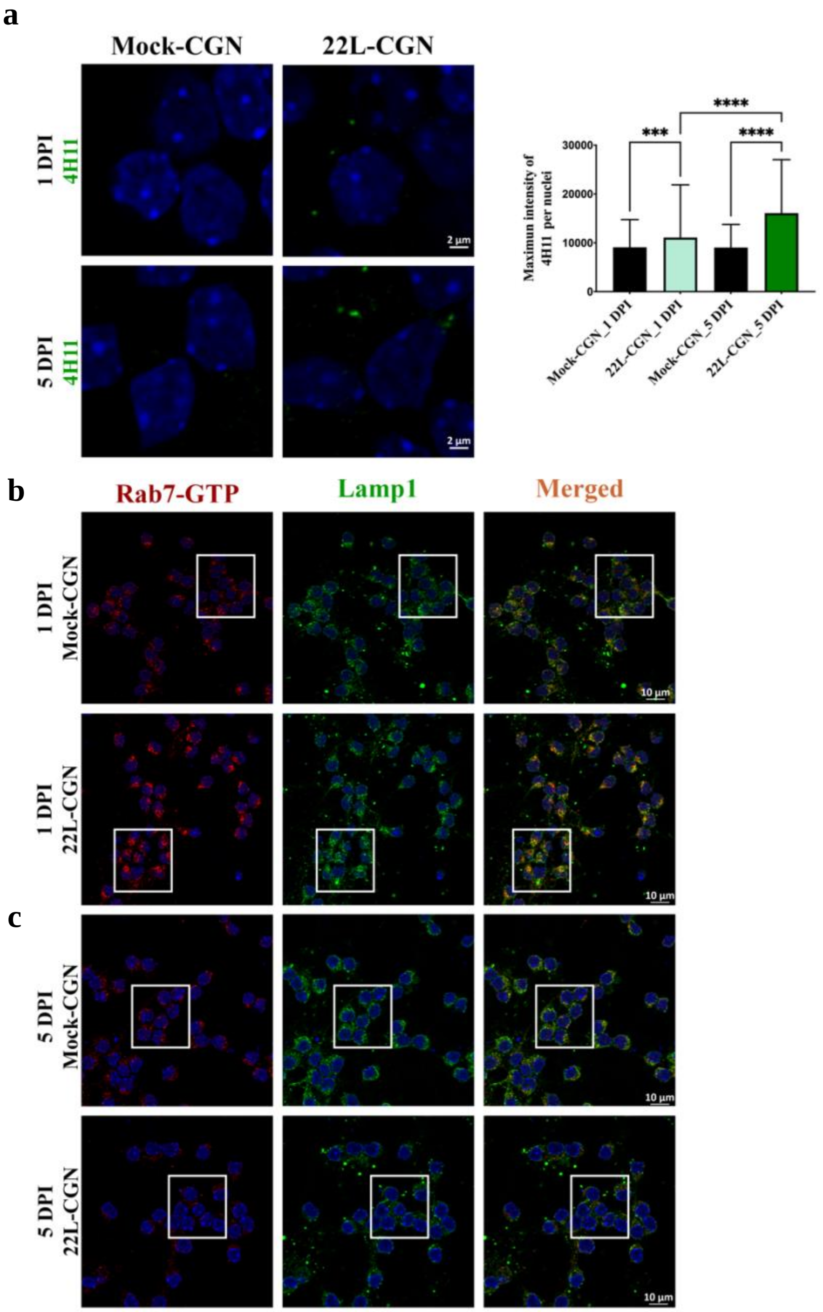
a
Mock-CGN
22L-CGN
1 DPI
4H11
5 DPI
4H11
2 µm
2 µm
Maximun intensity of
4H11 per nuclei
30000
20000
10000
0
***
****
****
Mock-CGN_1 DPI
22L-CGN_1 DPI
Mock-CGN_5 DPI
22L-CGN_5 DPI
b
Rab7-GTP
Lamp1
Merged
1 DPI
Mock-CGN
1 DPI
22L-CGN
10 µm
c
5 DPI
Mock-CGN
5 DPI
22L-CGN
10 µm

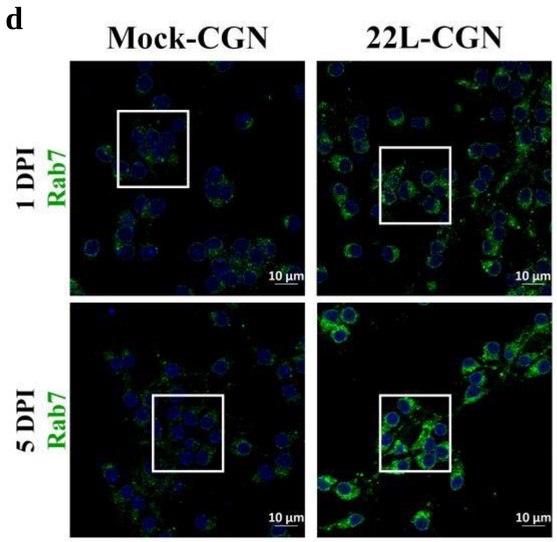

Figure SF2: Analysis of de novo prion infection primary cerebellar granular neurons and over-view images of Rab7-GTP staining in Figure 2. a) 22L-CGN cultures were treated with 6M GdnCl to detect PrP^{Sc} signals after probing with anti-PrP antibody (4H11) and confocal microscopy at1 DPI and at 5 DPI. Quantitative analysis of the PrP^{Sc} signal intensity was performed from 10 different fields of view which approximately amounts to 300 cells per experiment. Three independent experiments have been conducted with concurrent results with the one depicted here. Statistical significance was analysed using one-way ANOVA followed by post-hoc analysis using Šídák's multiple comparisons test. The error bars reflect standard deviation (***, *p* values <0.001; ****, *p* values <0.0001). Overview of the confocal images of the Rab7-GTP staining in primary cerebellar granular neurons at b) 1 DPI c) 5 DPI and d) Rab7 staining at 1 DPI and 5 DPI. Scale bars =10 µm, 2 µm.

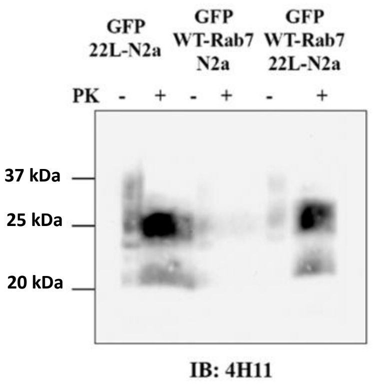

Figure SF3: Analysis of PrP^{Sc} levels in cells used for immuno-pull-down studies. N2a/22L-N2a cells that underwent immune-pull down were transfected with GFP tagged WT-Rab7 or GFP (as control) for 48 hours, then cells were lysed, and PrP^{res} levels analyzed by subjecting them to PK digestion and immunoblotting with anti-PrP antibody (4H11).

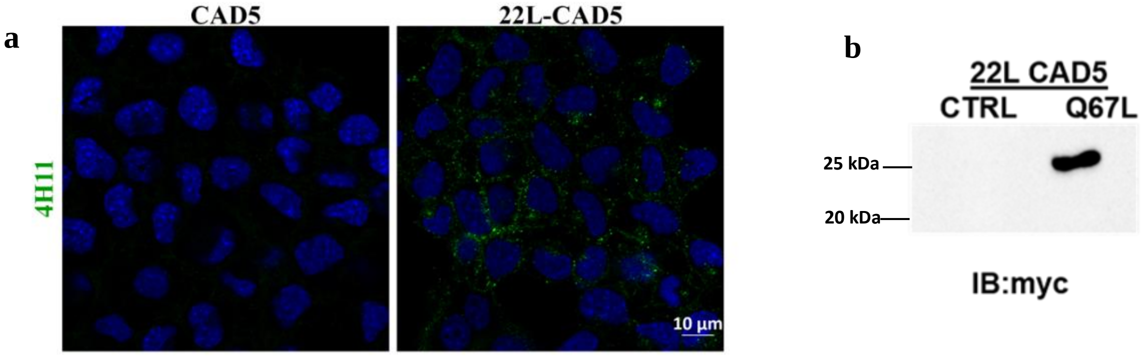

Figure SF4: Analysis of prion infection and transfection efficiency in CAD5 cells used in LDL-chase rescue experiment a) 22L-CAD5 cells used in the LDL-chase experiment, were treated with 6M GdnCl to detect PrP^{Sc} signals after probing with anti-PrP antibody (4H11). b) Analysis of the transfection efficiency of the myc-tagged constitutively active mutant of Rab7 (Q67L) in the 22L-CAD5 cells subjected to the LDL rescue experiment.

Supplementary Table 1: Summary of the statistical tests and p-values in the various experiments conducted in this study.

Figures	Parameters analyzed	Groups	Statistical tests	Post hoc analysis	p-values
Figure 1	Mean Rab7-GTP intensity per cell	N2a vs 22L-N2a	Unpaired t-test	N/A	<0.0001
	Mean Lamp1intensity per cell	N2a vs 22L-N2a	Unpaired t-test		<0.0001
	Colocalization coefficient	N2a vs 22L-N2a	Unpaired t-test		0.0006
Figure 2	Mean Rab7-GTP intensity per cell	Mock-CGN vs 22L-CGN (1 DPI)	Unpaired t-test	N/A	<0.0001
		Mock-CGN vs 22L-CGN (5 DPI)	Unpaired t-test		<0.0001
	Colocalization coefficient	Mock-CGN vs 22L-CGN (1 DPI)	Unpaired t-test	N/A	0.001
		Mock-CGN vs 22L-CGN (5 DPI)	Unpaired t-test		<0.0001
	Mean Rab7 intensity per cell	Mock-CGN_1 DPI vs. 22L-CGN_1 DPI	Ordinary One-way ANOVA	Turkey's multiple comparison test	<0.0001
		Mock-CGN_1 DPI vs. Mock-CGN_5DPI			0.0236
		Mock-CGN_1 DPI vs. 22L-CGN_5DPI			<0.0001
		22L-CGN_1 DPI vs. Mock-CGN_5DPI			<0.0001
		22L-CGN_1 DPI vs. 22L-CGN_5DPI			<0.0001
		Mock-CGN_5DPI vs. 22L-CGN_5DPI			<0.0001
Figure 3a	RILP normalized to GFP-Rab7	N2a vs 22L-N2a	Unpaired t-test	N/A	0.0229
Figure 3b	Mean vesicle distance closest to the nucleus	N2a vs 22L-N2a	Unpaired t-test		0.009
Figure 3c	Ub-Rab7 normalized to GFP- Rab7	N2a vs 22L-N2a	Unpaired t-test		0.0195
Figure 4a	Normalized Rab7 levels	Mock-BH vs 22L-BH	Unpaired t-test	N/A	0.0341
Figure 4b	Normalized RILP levels	Mock-BH vs 22L-BH	Unpaired t-test		0.0166
Figure 5a	Normalized filipin intensity	CAD5 vs 22L-CAD5	Unpaired t-test	N/A	<0.001
Figure 5b	Number of filipin puncta per cell	Mock-CGN vs 22L-CGN (5 DPI)	Unpaired t-test		<0.0001
Figure 5c	Fold change-HMGCoAr	CAD5 vs 22L-CAD5	Unpaired t-test		0.0068
	Fold change-LDLR	CAD5 vs 22L-CAD5	Unpaired t-test		0.0127
	Fold change-Sc4mol	CAD5 vs 22L-CAD5	Unpaired t-test		0.0154
Figure 5d	Fold Change-HMGCoAr (Lipid Depletion)	CAD5-- vs. CAD5+-	Ordinary One-way ANOVA	Šídák's multiple comparisons test	<0.0001
		CAD5+- vs. 22L-CAD5+-			<0.0001
		22L-CAD5-- vs. 22L-CAD5+-			0.0006

Figure 5f	Fold Change- HMGCoAR (Lipid Repletion)	CAD5+- vs. CAD5++	Ordinary One-way ANOVA	Šídák's multiple comparisons test	<0.0001
		CAD5++ vs. 22L-CAD5++			0.0104
		22L-CAD5+- vs. 22L-CAD5++			<0.0001
Figure 6	Colocalization coefficient (Rab5)	CAD5 vs 22L-CAD5	Unpaired t-test	N/A	0.0134
Figure 7	Colocalization coefficient (Rab6)	CAD5 vs 22L-CAD5	Unpaired t-test	N/A	0.0064
Figure 8	LDL Quantification	CAD5 vs. 22L-CAD5	Ordinary One-way ANOVA	Turkey's multiple comparison's test	<0.0001
		CAD5 vs. 22L-Q67L			0.0165
		22L-CAD5 vs. 22L-Q67L			<0.0001
	LDL Colocalization (Lamp1)	CAD vs. 22L-CAD	Ordinary One-way ANOVA	Turkey's multiple comparison's test	<0.0001
		CAD vs. 22L-Q67L			0.0062
		22L-CAD vs. 22L-Q67L			<0.0001
Figure 9a	Normalized cholesterol levels	N2a vs. 22L-WT	Ordinary One-way ANOVA	Turkey's multiple comparison's test	0.0005
		N2a vs. 22L-Q67L			0.3077
		N2a vs. 22L-T22N			0.1716
		22L-WT vs. 22L-Q67L			0.0041
		22L-WT vs. 22L-T22N			0.0070
		22L-Q67L vs. 22L-T22N			0.9692
Figure 9e	Normalized PrP^{Sc} levels	22L-EGFP vs. 22L-WT	Ordinary One-way ANOVA	Turkey's multiple comparison's test	0.9529
		22L-EGFP vs. 22L-Q67L			0.0048
		22L-EGFP vs. 22L-T22N			0.0002
		22L-WT vs. 22L-Q67L			0.0167
		22L-WT vs. 22L-T22N			0.0008
		22L-Q67L vs. 22L-T22N			0.5982
